# Advances in Preclinical In Vitro Models for the Translation of Precision Medicine for Cystic Fibrosis

**DOI:** 10.3390/jpm12081321

**Published:** 2022-08-16

**Authors:** Iris A. L. Silva, Onofrio Laselva, Miquéias Lopes-Pacheco

**Affiliations:** 1 S2AQUAcoLAB, 7800-194 Olhão, Portugal; 2 Department of Clinical and Experimental Medicine, University of Foggia, 71122 Foggia, Italy; 3 Biosystems & Integrative Sciences Institute, Faculty of Sciences, University of Lisbon, 1749-016 Lisbon, Portugal

**Keywords:** airway cells, cell lines, CFTR modulators, drug development, induced pluripotent stem cells (iPSCs), intestinal cells, organ-on-a-chip, organoids, personalized medicine, theratyping

## Abstract

The development of preclinical in vitro models has provided significant progress to the studies of cystic fibrosis (CF), a frequently fatal monogenic disease caused by mutations in the gene encoding the CF transmembrane conductance regulator (CFTR) protein. Numerous cell lines were generated over the last 30 years and they have been instrumental not only in enhancing the understanding of CF pathological mechanisms but also in developing therapies targeting the underlying defects in CFTR mutations with further validation in patient-derived samples. Furthermore, recent advances toward precision medicine in CF have been made possible by optimizing protocols and establishing novel assays using human bronchial, nasal and rectal tissues, and by progressing from two-dimensional monocultures to more complex three-dimensional culture platforms. These models also enable to potentially predict clinical efficacy and responsiveness to CFTR modulator therapies at an individual level. In parallel, advanced systems, such as induced pluripotent stem cells and organ-on-a-chip, continue to be developed in order to more closely recapitulate human physiology for disease modeling and drug testing. In this review, we have highlighted novel and optimized cell models that are being used in CF research to develop novel CFTR-directed therapies (or alternative therapeutic interventions) and to expand the usage of existing modulator drugs to common and rare CF-causing mutations.

## 1. Introduction

Cystic fibrosis (CF) is a life-threatening autosomal recessive disease affecting over 100,000 people worldwide [1,2]. It is caused by mutations in the gene encoding the CF transmembrane conductance regulator (CFTR) protein [3,4,5], a cAMP-dependent, phosphorylation-activated anion channel that transports chloride (Cl^−^) and bicarbonate (HCO_3_^−^) across the apical plasma membrane (PM) of several epithelial tissues, of which the lungs are the most affected organs [1,2]. In the airways, CFTR dysfunction or absence causes abnormal ion transport and dehydration of the epithelial surface liquid layer, leading to alterations in viscoelastic properties of mucus and its subsequent accumulation due to impaired mucociliary clearance [6]. As a consequence, people with CF (PwCF) face airway obstruction, recurrent infections and chronic inflammation that progressively promote tissue remodeling and decline of lung function, ultimately resulting in respiratory failure [6].

Managing CF has traditionally relied on therapeutics targeting signs and symptoms [7,8,9]. These consist of laborious physical and inhaled therapies and numerous daily remedies (mucolytics, antibiotics, anti-inflammatory drugs and pancreatic enzymes, among others) that together with the specialized multi- and interdisciplinary healthcare, and implementation of newborn screening programs led to significant improvements in PwCF’s longevity [7,8,9,10]. Nevertheless, although many PwCF are nowadays living into adulthood, their longevity is still limited and these individuals remain subjected to considerable clinical, psychosocial and economic burdens, which negatively affect their quality of life [2,11]. In order to further enhance their current longevity and steadily mitigate therapeutic burdens, CF must be treated beyond its symptoms by targeting the root cause of the disease, precluding thus the pathological cascade of events downstream of CFTR dysfunction.

The deletion of a phenylalanine at position 508 (F508del) is the most prevalent CF-causing mutation and found in ~70% of CF allele worldwide [2,12]. However, over 2100 *CFTR* genetic variants have been reported to date (CF Mutation Database, http://www.genet.sickkids.on.ca/, accessed on 22 July 2022). Although most are presumably to be pathogenic, only ~500 variants have the disease liability established (Clinical and Function Translation of CFTR Database, https://cftr2.org/, accessed on 22 July 2022 and CFTR-France Database, https://cftr.iurc.montp.inserm.fr/cgi-bin/home.cgi, accessed on 22 July 2022). All CF-causing mutations result in impaired CFTR-mediated Cl^−^/HCO_3_^−^ transport, but this occurs due to a range of distinct cellular/functional defects. Accordingly, CFTR mutations have been separated into six main functional classes, characterized by: (I) no synthesis of full-length protein, (II) defective protein folding and trafficking, (III) defective channel gating, (IV) decreased anion conductance, (V) decreased protein abundance, and (VI) decreased protein stability at the PM [2,12,13]. Despite many *CFTR* genetic variants remain to be characterized regarding their underlying cellular/functional defect(s), this classification system has been helpful to accelerate theratyping efforts as mutations within the same class are expected to be treated by the same therapeutic approach, although not necessarily by the same drug.

Since the discovery of the *CFTR* gene in 1989 [3,4,5], preclinical cell models have been playing a fundamental role in enhancing the understanding of CFTR biology at the genetic, biochemical and physiological levels [14,15]. In parallel, the implementation of cell-based high-throughput screening (HTS) assays has enabled the identification of specialized drugs targeting the primary defect(s) associated to CFTR mutations [12,13,16,17,18,19], which have been resulting in major therapeutic progress for PwCF. These CFTR modulator drugs are small molecules that can: (1) allow the insertion of an amino acid in a locus where a premature termination codon (PTC) was introduced into the CFTR mRNA (read-through agents or PTC suppressors); (2) enhance CFTR protein biosynthesis by stabilizing CFTR mRNA (amplifiers); (3) reestablish CFTR protein folding and its trafficking to the PM (correctors); (4) increase CFTR channel open probability (potentiators); and (5) augment the anchoring of the CFTR protein located at the PM (stabilizers). As most CF-causing mutations promote multiple structural defects on the CFTR protein, combination therapy is often required to rescue mutant CFTR at therapeutically relevant levels [2,12,13].

There are currently four CFTR modulator drugs licensed for clinical use by the Food and Drug Administration (FDA) and the European Medicines Agency (EMA). The approval of the first CFTR modulator—the gating potentiator VX-770 (ivacaftor)—occurred in 2012 after successfully improving lung function measured as percent of predicted forced expiratory volume in 1 s (ppFEV_1_) of PwCF carrying the CFTR gating mutant G551D in phase III clinical trials [20,21]. Despite its remarkable improvement of lung function (~10 ppFEV_1_), its approval was restricted to a small CF population (~4%). Subsequent clinical studies assessing either the solo effect of VX-770 or of the traffic corrector VX-809 (lumacaftor) were unable to improve lung function of PwCF homozygous for the CFTR folding mutant F508del [22,23]; however, combining these two drugs led to a significant, albeit modest, improvement in lung function (~4 ppFEV_1_) [24,25], which resulted in approval of this combination in 2015 and made this combination suitable for a greater number of PwCF worldwide (~40%). A newer corrector VX-661 (tezacaftor) used in combination with VX-770 was approved in 2018 but with similar limited therapeutic efficacy for F508del-homozygous PwCF [26]. Nevertheless, this corrector/potentiator combination therapy was also effective for those heterozygous for F508del with a second mutation resulting in CFTR residual function (i.e., classes IV–VI) [27], increasing thus to ~60% of PwCF who could benefit from modulator therapies. Only in late 2019, a triple combination composed of two correctors—VX-661 and VX-445 (elexacaftor)—plus the potentiator VX-770 was approved for clinical use. Indeed, this ‘highly effective’ modulator therapy demonstrated to significantly improve lung function (>10 ppFEV_1_) in phase III clinical trials not only in PwCF homozygous for F508del [28] but also in those carrying this mutation in one allele and a minimal function mutation (i.e., classes I/II) in *trans* [29]. Over this period, several label extensions have been approved (most by the US FDA) to uncommon CFTR mutations [13,15,30], and >85% of PwCF in North America, Oceania and various countries in Europe are currently eligible for at least one of these clinically approved modulator therapies.

Despite such remarkable accomplishments over the last decade, many PwCF carrying rare (and ultra-rare) mutations, as well as nonsense and splicing mutations, remain with no modulator therapy available. For instance, several countries, including Brazil, Israel and Italy, have ≥30% of PwCF carrying non-F508del mutations in both alleles [2]. Various novel therapies are currently in the experimental and early-stage clinical development (CFF Drug Development Pipeline, https://apps.cff.org/trials/pipeline/, accessed on 22 July 2022). These include not only CFTR-directed therapeutics but also targeting alternative channels/transporters to compensate for CFTR dysfunction and beyond [10,12,31]. Meanwhile, CF scientific community also continues to develop novel cell models to more efficiently predict clinical efficacy and responsiveness [14,32,33,34,35], since conventional clinical trial designs are underpowered and impractical for rare CFTR mutations due to the very low number of individuals [30,36,37]. Indeed, it is estimated that for >1000 *CFTR* variants there are ≤5 PwCF worldwide [12,15,37,38]. Accordingly, the current challenge of biomedicine for CF is not only identifying novel causative therapies but also in a personalized fashion so that every individual may achieve the greatest therapeutic benefits. In this review, we have highlighted novel and optimized cell models that are being used in CF research to identify novel CFTR-directed therapies (or alternative interventions) and to assess the utility of existing modulator drugs to common and rare CF-causing mutations.

## 2. Preclinical In Vitro Models

### 2.1. Cell Lines

The development of immortalized cell lines has provided substantial progress in CF research, particularly for the understanding of CFTR biology, characterization of common and rare CF-causing mutations (and exclusion of non-pathogenic CFTR variants), and identification of novel CFTR modulator compounds.

Multiple cell lines (non-human *vs*. human and non-epithelial *vs*. epithelial) have been generated and optimized to help in enhancing our knowledge of CFTR biology [14,15]. These include baby hamster kidney (BHK) [18,31,32], Chinese hamster ovary (CHO) [33,34,35,36], African green monkey kidney (Cos-7) [37,38,39,40], embryonic Swiss mouse (3T3) [17,35,41], human embryonic kidney (HEK) [36,41,42,43,44], Madin-Darby canine kidney (MDCK) [43,45,46], Fischer rat thyroid (FRT) [16,47,48,49] and CF bronchial epithelial (CFBE41o^−^) cell lines [32,43,49,50,51,52] (Table 1). These cells are easy to be cultured and expanded, making them suitable models for the usage in different methods and assays [14]. Furthermore, as most of these cells do not express endogenous CFTR, an initial characterization of the impact of variants on CFTR behavior can be obtained by either transiently or stably expressing CFTR cDNA. However, CFTR cDNA-based expression may not recapitulate elemental aspects of certain variants, including nonsense-mediated decay (NMD) for premature termination codons (PTCs) and canonical/non-canonical splice defects. For instance, G970R was initially classified as a CFTR gating mutant, based on cDNA expression, and demonstrated to be responsive to potentiators [36,40,53]. However, RNA analysis of patient-derived samples revealed that this mutant actually causes alteration of CFTR splicing with exon skipping and, consequently, is not rescued by VX-770 alone [54,55], which corroborates with the lack of in vivo response in PwCF [56]. A feasible solution to overcome such misclassification in engineered cell lines is the expression of these types of variants in mini-gene systems [43,57,58,59]. The usage of these cell models is also valuable in situations where there is a low availability or accessibility of patient-derived samples (tissue or primary cells) for research purposes and when researchers aim at investigating CF genotypes combining two different rare CFTR mutations (one in each allele) or the effect of a complex allele.

The HTS of compound libraries using cell line-based assays have been the mainstay to identify CFTR-directed modulators for subsequent development [13,16,17,18,19]. However, it is highly recommended that most promising compound(s) identified in cells heterologously expressing CFTR construct should be further validated in a native system. Indeed, the existing cell models have some liabilities due to immortalization processes that can cause genome instability, karyotypic alterations and modifications in gene expression [14,60,61]. Furthermore, cell background has a strong influence on the pharmacological rescue of CFTR mutations, as it has been previously demonstrated for F508del-CFTR when the same compound is assessed in different cell models [31,52,62,63,64]. Validation of several hit compounds (or combinations thereof) on primary cells (i.e., human bronchial epithelial (HBE) cells) may be nevertheless unfeasible, as these are a limited resource and usually require invasive procedures to be obtained (bronchoscopy or explants from lung transplantation). To overcome this, the usage of immortalized human airway cells may be a practical solution to restrict the number of putative hit compounds before moving forward to the validation in primary cells. Accordingly, the immortalized bronchial epithelial CFBE41o^−^ cell lines are considered a more physiologically relevant model in the context of CF and have a more stringent cell quality control system in comparison to non-human and non-epithelial cell lines [51,62]. These cells have been extensively used in CF research and several methods have been optimized to facilitate heterologous expression of CFTR variants on CFBE41o^−^ cell lines, including incorporation of multiples copies to enable transgene overexpression, constructs with inducible promoters or a single recombinant target site for transgene integration [19,32,44,50,65].

The immortalized human bronchial epithelial 16HBE14o^−^ cell lines endogenously expressing WT-CFTR have also been used as a positive control in CF studies [60,66]. More recently, CRISPR/Cas9-mediated gene editing has been exploited to generate isogenic, homozygous 16HBE14o^−^ cell lines harboring CFTR variants in the native genomic context [67]. These cells may have utility not only for assessing modulator drug efficacy [67,68,69,70,71] but also for gene-editing therapeutic approaches targeting the CFTR gene [72,73]. Nevertheless, although it has been reported that these 16HBE14o^−^ gene-edited cells endogenously expressing CFTR variants have demonstrated steady, reproducible responses in different assays up to 25 passages after cryopreservation [67], it remains unclear whether they may lose certain properties in even higher passages (e.g., polarization ability) or whether their physiology may fully reflect that of primary airway cells.

Despite cell lines are unable to predict therapeutic responses in PwCF at an individual level, they have been useful in supporting drug discovery and development for common and rare CF-causing mutations [2]. Indeed, the FDA has licensed label extension of clinically approved CFTR modulators to several additional mutations based on data from FRT cell lines heterologously expressing mutant CFTR cDNA [13,15,30] with subsequent clinical studies confirming the therapeutic benefits for some of these mutations [56,74,75]. Furthermore, a study has paired in vitro measurements of CFTR function in either FRT or CFBE41o^−^ cell lines stably expressing CFTR variants with clinical and genetic data from the CFTR2 database, and demonstrated that there is a strong correlation between CFTR function and sweat Cl^−^ levels [76]. A statistically significant correlation, albeit modest, was also observed for CFTR function with pancreatic status and lung function [76].

### 2.2. Patient-Derived Samples

There has been a growing interest in assessing the efficacy of modulator drugs on ex vivo patient-derived samples over the last years since these cell models may recapitulate several features of parental organs and might predict clinical efficacy and responsiveness [77,78,79]. Nevertheless, all of these patient-derived cell-based models require specialized technicians and standard operating protocols to ensure reproducibility among different laboratories. It is also particularly noteworthy that an inter-variability in responses to CFTR modulator therapies has been reported even among individuals carrying the same CF genotype [20,24,26,28,80]. Accordingly, these models may be useful to further understand the impact of non-CFTR genetic factors (e.g., modifier genes) on each individual’s disease [81]. Finally, rare and ultra-rare CFTR mutations are often found in ethnic and racial minority populations, which are usually not included in conventional clinical trials [82]. By using these cell models, it is possible to identify those PwCF that may clinically benefit from available modulator therapies. We have further described these models below and listed key advantages and limitations of each (Table 2).

#### 2.2.1. Lung/Airway Models

- Primary Airway Cells in Planar Cultures

**Figure 1:**
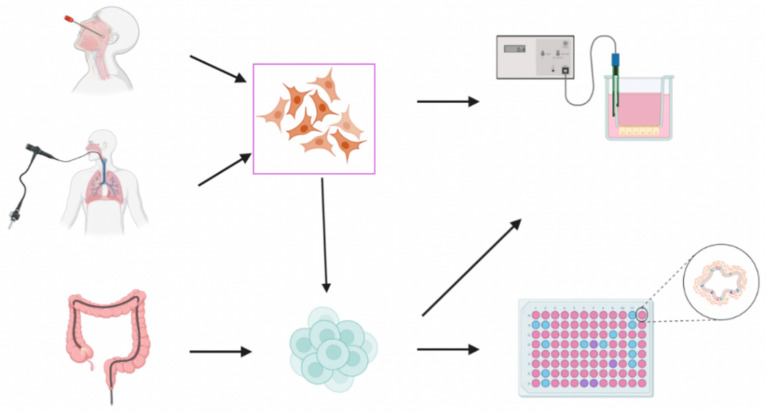
**Overview of available strategies to generate patient-derived models.** (**Top**) Well-differentiated airway epithelium cultures can be obtained from nasal or bronchial brushing and cultured at the air-liquid interface (ALI). The 2D ALI cultures could be used as a preclinical tool to test novel small molecules by electrophysiological studies. These cells can also be cultured in a 3D matrix to increase the throughput/scalability as airway organoids. (**Bottom**) The 3D intestinal organoids are obtained from rectal biopsy and could be used for high-throughput screening to investigate novel therapies. These organoids can also be grown in 2D monolayers to assess CFTR rescue and function by traditional electrophysiological measurements.

Primary HBE cells derived from bronchial brushing or explant lungs of PwCF have been considered the gold standard to validate the efficacy of CFTR modulators in vitro [19,83,84,85] (Figure 1). Well-established protocols has been used to conditionally reprogram, expand and maintain primary HBE cells to higher passage numbers, while avoiding their premature cellular senescence and squamous transformation [85,86,87,88,89]. Accordingly, these cells acquire stem-like features but are still able to differentiate into the several airway epithelial cell types on porous membrane filters at air-liquid interface (ALI) conditions, mimicking thus the polarized, pseudostratified epithelia of in vivo airways [85,89,90]. Indeed, these cells have had a fundamental role not only in in the assessment of modulator therapies but also in enhancing the current understanding of CFTR biology in the respiratory tract. In electrophysiological measurements, such as Ussing chambers (where electric currents are measured based on the transport of ions), HBE cultures from PwCF exhibited a defective CFTR-dependent anion transport [83,84,89,91], while reduction in surface liquid layer and ciliary beat frequency, and mucostasis were also observed by optical techniques [92,93,94].

Despite the adoption of conditionally reprogramming method, the availability and accessibility of primary HBE cells remain relatively poor as a highly invasive procedure is still required to obtain the cells, which limits their usage in precision medicine for CF, particularly for testing novel therapeutic approaches targeting rare CF-causing mutations. On the other hand, human nasal epithelial (HNE) cells can be harvested by nasal brushing or scaping in minimal invasive procedures [95,96] (Figure 1), and be subsequently cultured under conditionally reprogramming conditions using the same protocols as for HBE cells to expand their availability and lifespan without affecting CFTR expression and function [88,97,98]. Although certain epithelial cell types can differ along the respiratory tract (i.e., nasal cavity, bronchi and bronchioles) [99,100,101,102], several studies have indicated that HBE and HNE cells differentiated at ALI exhibit similar morphological and functional properties, and response to inflammatory mediators [95,103,104,105,106]. Electrophysiological measurements of HBE and HNE cells from the same individual with CF demonstrated similar CFTR-dependent ion transport [79,95]. HNE cultures have also been used assess the efficacy of gene therapy [107,108,109,110] and to measure effects downstream of CFTR dysfunction, including reduction in air surface liquid height, pH and mucociliary transport rate [111,112]. Furthermore, HNE cells have been considered a relevant surrogate for HBE cells in pre-clinical studies, since both cell types have responded to CFTR modulator drugs in a similar fashion [79,95,98,113,114].

Recent studies have established correlations between measurements of CFTR function in HNE cultures and clinical features/biomarkers to potentially predict therapeutic outcomes by modulator therapies. A strong correlation was observed between VX-770-induced CFTR-dependent currents in HNE cultures and changes sweat Cl^−^ levels and ppFEV_1_ of PwCF carrying either R117H- or G551D-CFTR [115]. In N-of-1 trial series, CFTR-dependent Cl^−^ secretion was increased only in HNE cultures of three PwCF who also exhibited a decrease in sweat Cl^−^ levels after VX-770 therapy [116]. Furthermore, responses from HNE cultures carrying either F508del/F508del [79,103] or rare CF genotypes [98,117] demonstrated a correlation with changes in sweat Cl^−^ levels, ppFEV_1_ or intestinal current measurement (ICM), but not with nasal potential difference (NPD, a functional assay that indirectly assesses ion transport in the nasal epithelium in vivo) after VX-809/VX-770 or VX-661/VX-770. Although further studies should be performed to establish correlations between in vitro measurements and individual specific clinical outcomes, HNE cultures have demonstrated to be a useful model for theratyping.

- Airway Organoids/Spheroids

Several protocols have been recently optimized to develop airway organoids/spheroids derived from nasal polyps, nasal or bronchial brushing, explanted lungs, cells from bronchoalveolar lavage fluid or differentiated from induced pluripotent stem cells (iPSCs) [113,114,118,119]. This model is particularly relevant to provide a high-throughput measurement of CFTR function for either drug or gene therapy studies in primary airway cells, since the classical assessment in HBE and HNE cells is based on short-circuit current recordings, which is a low-throughput technique. In contrast to ALI monoculture models, airway organoids are embedded in a three-dimensional matrix composed of several structural proteins, including fibronectin, laminin and collagen, which provide physiological and architectural support for their growth and expansion [120,121]. This model has been used to study several chronic respiratory diseases, including but not limited to CF, viral infections and lung cancer. Airway organoids might thus represent the complex three-dimensional microenvironment of the airways by promoting differentiation of various cells types to physiologically resemble the diverse structural branching within the respiratory tract [120,121,122]. This model also allows for assessment of several channels’ function (not only CFTR but also other channels, such as ENaC and TMEM16A) and provides a complementary measurement of ion/fluid transport to ALI cultures.

In CF studies, airway organoids were initially developed from HBE and HNE cells, and used to assess CFTR-dependent fluid secretion and thus discriminate CF cultures from non-CF ones [113,114]. This model recapitulates several features of in vivo airway epithelia, including expression of tight junctions, cilia and mucins, and CFTR function [123]. Furthermore, responses from Ussing chamber measurements of HNE cultures were demonstrated to correlate with forskolin-induced swelling (FIS) of airway organoids [106]. The FIS of organoids is a microscope-based functional assay that enables to quantify CFTR-dependent fluid secretion after stimulation of CFTR activity by forskolin. Accordingly, there is an increase in the organoid size/swelling when CFTR is activated/rescued.

Pharmacological rescue of F508del-CFTR was demonstrated by treating airway organoids with VX-809/VX-770 [113,114]. In parallel, CFTR rescue was also demonstrated by VX-809/VX-770, VX-661/VX-770 or VX-445/ VX-661/VX-770 in a range of airway organoids from individuals carrying rare CF genotypes [106,118]. A significant correlation was also observed between organoid swelling (baseline and modulator rescued conditions) and clinical measurements (sweat Cl^−^ levels and ppFEV_1_) [106]. Nevertheless, this model is still less developed compared to intestinal organoids (the most advanced three-dimensional in vitro model in CF so far, see next section for further details), thus only a few studies are available. Some disadvantages have also been reported, such as a greater variability in results compared to that observed in ALI cultures [113,114] and the slower growth rate compared to intestinal organoids, which reduces the amount of material available for testing and, consequently, decreases the number of drugs (or combinations thereof) and replicates possible from the same individual’s sample.

#### 2.2.2. Gastrointestinal Models

- Rectal Biopsies

Although the respiratory tract is the most affected in CF, it is well known that CFTR is highly expressed in gastrointestinal cells, which make them valuable models to investigate CFTR function and modulation [124,125]. Furthermore, in contrast to the airways, CFTR is the dominant Cl^−^ channel responsible for Cl^−^ and fluid secretion in the human colon [124,125,126,127]. Finally, the intestine is not affected as the airways by chronic inflammation and infection with CF pathogens or suffers organ damage and remodeling, which are factors that may significantly affect CFTR channel function independent of the basic molecular defect of CFTR mutations [128].

Along these lines, ICM has been developed to quantify CFTR-dependent Cl^−^ secretion in native intestinal tissues. ICM is an ex vivo method in which a freshly excised rectal biopsy is mounted in an Ussing chamber and CFTR-mediated Cl^−^ responses to agonists that increase cAMP are measured [127,129,130]. By using ICM to characterize CFTR dysfunction, protocols were developed to differentiate between impaired CFTR function in intestinal tissues from PwCF *vs*. normal CFTR function in healthy control subjects [131,132,133]. In addition, protocols were further refined to enable the classification of CF into (I) lack of detectable CFTR function and (II) residual CFTR function [130,131,132,134]. Based on these results, ICM was established as a diagnostic and prognosis test to aid the establishment or refuting a diagnosis of CF, when sweat tests are unclear and/or when the functional consequence of rare or newly detected CFTR mutations are unknown [135].

With the advent of ‘highly effective’ CFTR-directed therapeutics in the clinics, and the fact that clinical trials of CFTR modulators demonstrated heterogeneous responses in clinical outcomes as well as sweat Cl^−^ levels among individuals with the same CF genotypes [24,26,28,80], additional sensitive biomarkers of CFTR function are critical to understand the degree of functional rescue of CFTR mutations by different CFTR modulator drugs, both at the level of CFTR genotype groups as well as individual’s responsiveness. In this context, ICM was also demonstrated to detect improvement of CFTR function when intestinal tissue biopsies were obtained from PwCF that were treated systemically with CFTR modulators [136,137,138,139], namely studies demonstrating that ICM is sensitive to detect in vivo activation of CFTR in PwCF carrying a G551D mutation and treated with VX-770 [136] or those homozygous for F508del and treated with VX-809/VX-770 [137,138] or with VX-445/VX-661/VX-770 [139]. Such fact indicates that ICM is a sensitive biomarker of CFTR function that could to facilitate and enhance precision therapy for PwCF [9,128,140].

- Intestinal Organoids

Intestinal organoids can be isolated from different regions of the human intestine. In the CF field, the most commonly used organoids are isolated from rectal biopsies, where the stem cell’s niche in the intestinal crypts can be isolated and cultured in a matrix with specific culture medium that provide important growth factors to allow for the expansion and stemness maintenance of these cells in order to form the three-dimensional in vitro structure named intestinal organoids [141,142] (Figure 1). These cells can be expanded over long periods without losing their stemness and biobanked to assess the efficacy of current and future modulator drugs [77,141,143]. In the context of CF, CFTR protein expression/function determines the morphology of these organoids by inducing swelling of non-CF organoids through salt and water accumulation in the lumen surrounded by a cellular layer, while organoids from PwCF have no lumen [141]. When CFTR is rescued by any modulator drugs, there is an increase in the organoid area, which can be quantified as an indirect measurement of CFTR activity by the FIS assay [142]. Recent studies have also used the FIS assay of intestinal organoids to assess novel gene therapeutic approaches [144,145].

CFTR-dependent fluid secretion properties in intestinal organoids are reflective of or related to CFTR function across several tissues. Indeed, CFTR rescue by modulator drugs in FIS of intestinal organoids was correlated with results from other cell types from the same individual, namely rectal biopsies and nasal epithelial cells [106,146]. A direct comparison of FIS with sweat Cl^−^ levels also revealed that the former has a strong correlation with disease severity when compared to the latter, which is the gold standard biomarker of CF disease and commonly used endpoint to measure efficacy of CFTR modulators [77,78,147,148]. Moreover, the FIS assay facilitates repeated measures and appears to be completely CFTR dependent, which reduces the impact of technical and other (non-CFTR) biological variability [141,147,148], whereas a higher heterogeneity in sweat Cl^−^ levels can occur due to technical issues and non-CFTR dependent biological factors [147]. FIS assay of intestinal organoids can also provide some advantages compared to other biomarkers used in CF diagnosis, such as NPD and ICM. Although NPD has been used to discriminate between PwCF and healthy controls, its ability to accurately establish differences in disease severity is limited [149,150]. On the other hand, ICM measurements are more sensitive and have a larger dynamic range than NPD, but generation of a large dataset with repeated measures is hampered by the need for fresh and of good quality rectal biopsies [151]. Finally, the feasibility of assessing combinations of multiples compounds to rescue mutant CFTR was recently demonstrated in intestinal organoids [152]. As an example, a quintuple combination composed of a read-through agent (ELX-02), an NMD inhibitor (SMG1i), two correctors (VX-445 and VX-661) and a potentiators (VX-770) was used and demonstrated to significantly rescue CFTR function of nonsense mutations for most analyzed intestinal organoids, although with variable response between donors [152].

Results from FIS assay have also been compared with measurements of biomarkers/clinical parameters in order to establish a reliable prediction of individual responsiveness to CFTR modulators. In children with CF, FIS of intestinal organoids demonstrated to correlate well with sweat Cl^−^ levels and ICM, enabling their stratification according to disease severity [148]. A consistent correlation was also observed among FIS of intestinal organoids, ppFEV_1_ and body mass index of adults with CF carrying F508del in both alleles, despite some variability in clinical features [153]. After CFTR modulator therapies, responses from intestinal organoids were demonstrated to correlate with ICM measurements, reduction in sweat Cl^−^ levels and improvements in ppFEV_1_ [77,78,154], suggesting its feasibility to guide label extension or compassionate use for PwCF with rare genotypes [78,155]. Nevertheless, no significant correlation was observed by FIS of intestinal organoids and changes in clinical measurements (sweat Cl^−^ levels, NPD, ICM and ppFEV_1_) of F508del-homozygous PwCF treated with VX-809/VX-770 [156]. Such divergence may be in part related to the modest effect of VX-809/VX-770 (or even VX-661/VX-770) and the small sample size used in that study, suggesting that assessment in a larger group of PwCF and of more effective CFTR modulators should be further investigated.

- Intestinal Organoids-Derived 2D Monolayers

More recently, intestinal organoids have been used to generate two-dimensional monolayers grown on porous membrane filters to assess CFTR rescue and function by traditional electrophysiological measurements. Such model may also be a valuable tool to: (1) measure ion transport of alternative channels; (2) modulate expression of ion channels/transporters via differentiation culture conditions; and (3) assist in diagnosis and precision medicine testing. These facts are of particular relevance for PwCF carrying rare genotypes, who are unlikely to participate in conventional clinical trials, and to investigate CFTR-related disorders.

Intestinal organoids-derived 2D monolayers can provide an easy access to the apical, lumen-facing membrane and the opportunity to directly assess CFTR-dependent ion transport by Ussing chamber measurements [157,158]. Indeed, CFTR activity measured on these monolayers carrying distinct CF genotypes demonstrated a good correlation with results from donor-matched native ICM and FIS of 3D intestinal organoids [157,158]. Compared to FIS assay, Ussing chamber measurements in 2D monolayers yet allow to measure CFTR-mediated Cl^−^ and HCO_3_^−^ transport separately [158]. Such fact holds great importance since modulator drugs that are able to restore CFTR folding demonstrated to differently impact on CFTR-dependent Cl^−^ vs. HCO_3_^−^ transport [159,160]. Furthermore, 2D monolayers have a broader dynamic range, enabling thus to segregate responses from none/very low to high residual CFTR function [158,161] and use WT-CFTR currents as a reference value to compare efficacy of modulator drugs on different CFTR mutations. Comparing the performance of difference model systems is the current challenge to predict therapeutic responsiveness for real life cases. In this context, multiple variables such as dynamic range of the assay, sensitivity and specificity to detect CFTR-mediated ion transport should be considered in translational and clinical research.

### 2.3. Induced Pluripotent Stem Cells (iPSCs)

**Figure 2:**
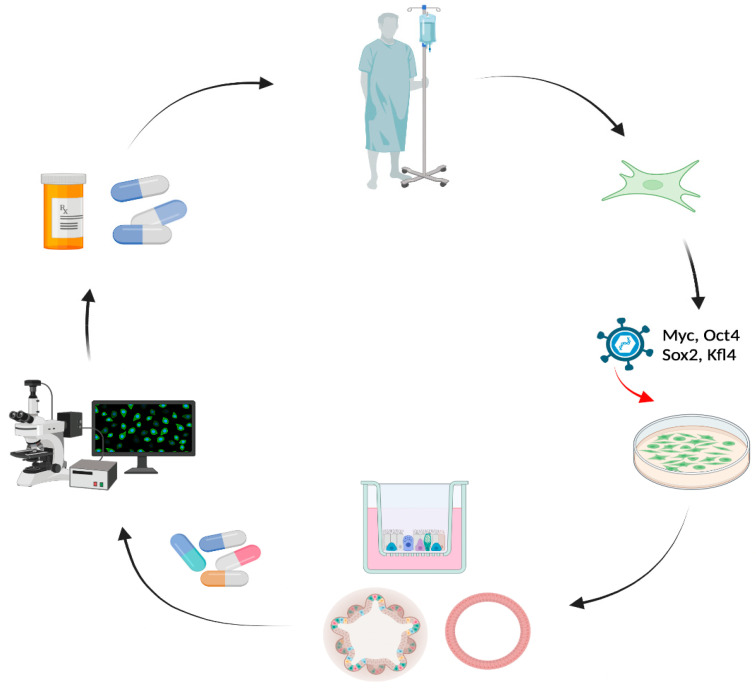
**Human induced pluripotent stem cells (iPSCs) as a preclinical tool for personalized medicine in Cystic Fibrosis.** iPSCs can be generated from adult somatic cells (i.e., skin fibroblast, peripheral blood mononuclear cells, etc) by introducing the reprogramming genes (c-Myc, Oct4, Sox2 and Kfl4). Using efficient protocol to differentiate, iPSCs could be used to generate iPSC-derived lung, cholangiocytes or intestine epithelial cells to test small molecules to restore the impaired CFTR function.

iPSCs have been used as a preclinical model to understand the CF pathological mechanisms in different tissues. iPSCs are generated by reprogramming somatic cells to regain their pluripotency using viral vectors that introduce four transcription factors Oct3/4, Sox2, Klf4 and c-Myc in the culture conditions (Figure 2). These cells are then able to differentiate into several cell types, including lung and intestinal epithelial cells, neural cells and cardiomyocytes using specific protocols [162]. Due to their capacity for infinite expansion and differentiation virtually in any cell type, iPSCs are used in developmental biology, to understand the pathophysiology of diseases, and well as for drug discovery and cell-based therapy. Several research groups have generated differentiation protocols for iPSCs to generate airway and intestinal epithelial cells, and cholangiocytes, which are the main tissues affected in CF [163]. Accordingly, iPSCs have a potential use as CF patient-derived tissues in order to assess CF therapy in different cell types from the same individuals and as an in vitro model to screen novel therapeutic interventions.

There are several differentiation protocols established to generate proximal or distal lung cells from iPSCs providing a renewable source of CF patient-derived tissue that can be exploited for HTS of novel CFTR modulators, theratyping and beyond [164,165,166,167,168]. The benchmark for a successful differentiation of iPSCs to lung tissue is the functional expression of CFTR. The first protocol showing CFTR function in iPSC-derived airway cells was demonstrated by the FLIPR membrane potential assay, which allows for quantification of CFTR function by using a voltage-sensitive fluorescent dye [164,169,170]. Additionally, other groups demonstrated the measurement of the CFTR channel function by traditional electrophysiological techniques (i.e., whole-cell patch clamp and Ussing chamber measurements) [167,171]. In particular, it has been demonstrated that the functional defect of F508del-CFTR in airway epithelial cells from CF iPSCs was restored by CFTR correctors (i.e., C18, VX-809, VX-661) [164,170,171]. Moreover, the iPSC model was used to test novel therapeutic strategies to restore nonsense mutations (i.e., W1282X) and splicing mutations (i.e., I1234V) on iPSC-derived lung progenitor cells [172], reinforcing that airway epithelial cells from CF iPSCs can be used as a preclinical tool for precision medicine. More recently, iPSCs from PwCF carrying class I-III mutations demonstrated to serve as multimodal platforms for drug screening by Ussing chamber measurements and FIS assay [173].

It has also been demonstrated that iPSCs could be used to generate airway lung organoids to measure CFTR function by the FIS assay [165,174]. Interestingly, CF iPSC-derived lung organoids showed a poor forskolin-induced swelling, which was restored by gene editing to correct the F508del mutation to WT-CFTR [165]. These data demonstrate the applicability of this model in CF research, and that gene editing represents a very promising technology for correcting CFTR mutations [175,176,177]. Recently three different approaches were employed to correct the F508del mutation in iPSC-derived airway epithelial cells. These approaches include CRISPR/Cas9, Zinc-finger nucleases (ZFNs) and transcription activator-like effector nucleases (TALENs) [171,178,179]. In particular, after gene correction, iPSCs were differentiated to iPSC-derived airway epithelial cells expressing WT-CFTR located at the apical PM and its function was measured by patch-clamp or Ussing chamber studies.

Another established model for precision medicine in CF is the iPSC-derived intestinal cells [169,180,181,182]. The differentiation protocol for generating proximal intestine in a 2D model has shown its utility in identifying novel CFTR correctors by using an HTS assay [169,183]. Moreover, it has been recently demonstrated that iPSC-derived intestinal organoids can be employed to study other relevant membrane proteins (i.e., ENaC and electrogenic acid transporters) in addition to the CFTR channel [184]. Therefore, this novel method allows the screening of novel CFTR modulators, as well as small molecules that are able to modulate other channel/transporters relevant in CF disease.

Using specific protocols, iPSC may provide the opportunity to study CFTR function and small molecules screening in relevant tissues for CF that are not easily accessible for sampling (e.g., liver and pancreas). For instance, iPSC-derived pancreatic organoids from two PwCF demonstrated a decrease in FIS compared to iPSC-derived pancreatic organoids from healthy control individuals [185]. Moreover, the authors showed that CF pancreatic organoids could be used to screen CFTR modulators and mRNA-mediated gene therapy [185].

In addition to the aforementioned cell types, iPSCs have been differentiated to generate cholangiocytes, which express CFTR and are responsible to alter bile composition in the biliary ducts [186]. Indeed, CFTR demonstrated to be functional in non-CF iPSC derived cholangiocytes, while it was dysfunctional in iPSC-derived cholangiocytes expressing F508del-CFTR [186,187]. Nevertheless, CFTR modulators demonstrated to rescue functional expression of F508del-CFTR in this in vitro model [186,188]. Moreover, a recent report described a highly efficient protocol to develop mature, ciliated cholangiocytes [189]. In particular, the authors demonstrated the utility of these mature iPSC-derived cholangiocytes for testing novel CFTR modulators by HTS and the role of cilia in cholangiocytes development and function [189]. Such facts highlight the importance of iPSC-derived model to provide insight into CF and CFTR-related disorders, and they may be useful to predict disease progression and therapeutic benefits.

### 2.4. Organ-on-a-Chip System

**Figure 3:**
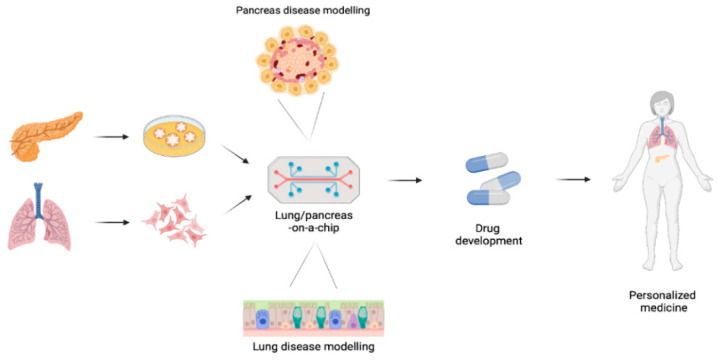
Graphical representation of lung and pancreas on-a-chip development for disease modeling and drug development for personalized medicine. To generate pancreas disease modeling, pancreatic ductal epithelial cells and pancreatic islets cells can be seeded into microfluid systems. This pancreas on-a-chip may be used to study CFTR-related disorders and drug development in CF. Moreover, airway or alveolar epithelial cells with pulmonary microvascular endothelial cells can be seeded into the device to generate lung-on-a-chip in order to investigate the in vivo environment of human airways and drug development in CF.

Although considerable advances have been made in the development of in vitro models as surrogates of tissues, these model systems do not recapitulate the tissue–tissue interface between endothelium and parenchymal tissues with critical transport of fluids, nutrients, immune cells and growth factors, among others [190]. In this context, advanced systems continue to be developed in order to more closely resemble human organ physiology for disease modeling and drug testing. These include organ-on-a-chip devices that are microfluidic systems constructed to integrate two cell culture chambers (e.g., epithelial cells from a patient interfaced with human microvascular endothelium) separated by a porous matrix-coated membrane [191,192] (Figure 3). In this micro-physiological systems, human cells are grown inside particular devices that stimulate the various 3D tissue interfaces, electrical stimuli and the organ function. Accordingly, this technology can recreate the smallest functional unit of any human organ [190].

Organ-on-a-chip technology enables to progress from traditional planar monocultures to more complex 3D co-culture systems, while also allow for the incorporation of environmental conditions, such as hypoxia, inflammation, infection and mucus plugging [193,194,195,196]. In a microvascularized human lung-on-a-chip platform, an increase in neutrophil migration to the vascular network was observed when CF HBE cells were seeded in the epithelial layer interface [193]. A favorable environment for *Pseudomonas aeruginosa* growth, mucus accumulation and enhanced secretion of inflammatory cytokines were also reported in a CF lung-on-a-chip system, demonstrating the ability of this model to recapitulate several important clinical features of the disease [194]. In other studies, human airway function, structure and inflammatory responses to allergic asthma and chronic obstructive pulmonary disease were successful modeled in lung-on-a-chip systems [195,196]. This microfluidic-based model holds particular relevance for CF, since small rodent models do not recapitulate most detrimental aspects of the lung disease [197,198]. In parallel, a CF pancreas-on-a-chip platform was created to assess the cell-cell communication by interfacing pancreatic ductal epithelial cells and pancreatic islets from the same individual [199]. CFTR is only expressed in the pancreatic ductal epithelial cells and its dysfunction led to a significant reduction of insulin secretion in islet cells [199]. Accordingly, this model can be useful not only for testing CFTR modulator therapies but also to better understand and monitor CFTR-related disorders.

## 3. Outlook and Conclusions

Major advances have been achieved in treating CF over the last decades. Preclinical in vitro models have been fundamental tools to gain insights into CF pathophysiology at the molecular, cellular and tissue levels, and to establish the relationship between CFTR mutations and disease phenotypes. These models have also paved the way for the development of CFTR-directed therapeutics and for the search for alternative or complementary therapeutic interventions.

With the introduction of CFTR modulator drugs into clinical practice, novel models and assays using patient-derived samples (i.e., bronchial, nasal and rectal tissues) have emerged and they provide a translational perspective to assess in vitro drug efficacy and to potentially predict in vivo therapeutic responsiveness. Indeed, such models hold particular relevance in CF not to identify which drug(s) may provide the greatest therapeutic benefits for each individual but also to expand the license of available (and novel) modulator drugs to individuals carrying rare CF genotypes that are responsive. Moreover, a survey demonstrated good acceptance of adults with CF and parents of children with CF to the usage of patient-derived samples for theratyping, and a preference for the least invasive site for tissue harvesting (i.e., nasal cavity rather than lower respiratory tract or rectum) [200].

In order to more closely recapitulate human organ physiology, more advanced models continue to be developed for disease modeling and drug testing. These systems enable to study intercellular communication and incorporate downstream consequences of CFTR dysfunction in the same platform, including inflammation, infection and mucus burden. It is also noteworthy that advanced models, such as iPSCs and organ-on-a-chip systems, require extensive training with specialized research teams in order to ensure an adequate protocol for implementation and standardization. Therefore, it is imperative to develop precise in vitro tools to establish strong correlations between CFTR measurements in these models and clinical features/biomarkers, and to assure that precision medicine will become available for all PwCF.

## Figures and Tables

**Table 1:** Cell lines commonly used in CF research.

Cell Type	Examples	Most Frequent Uses
Non-human non-epithelial	BHK, 3T3	Extensively used in initial HTS assays Characterization of CFTR variants and CFTR biology
	CHO, Cos-7	Characterization of CFTR biology Assessment of common and rare CFTR variants (transient expression)
Human non-epithelial	HEK	Assessment of common and rare CFTR variants (transient and stable expression)
Non-human epithelial	MDCK	Characterization of CFTR biology
	FRT	Extensively used in HTS assays Assessment of common and rare CFTR variants (transient and stable expression) Label extension of clinically approved CFTR modulators (FDA only)
Human epithelial	CFBE41o^−^	Extensively used in HTS assays Characterization of CFTR biology Assessment of common and rare CFTR variants (transient and stable expression)
	16HBE14o^−^	Assessment of common and rare CFTR variants in the native genomic context

**Table 2:** Key advantages and limitations of patient sample-derived models to assess CF therapies.

Model	Advantages	Limitations
HBE cells in monolayer culture	Well-established protocols to assess efficacy of CFTR modulators Recapitulate lung disease in CF Assessment of downstream consequences of CFTR dysfunction (e.g., mucociliary transport rate) Potential usage to investigate modulation of alternative channels/transporters	Requirement of invasive procedures to be harvested Low availability/accessibility, particularly for rare CF genotypes Low throughput/limited scalability to assess multiple compounds (or combinations thereof)
HNE cells in monolayer culture	Minimal invasive procedures to be harvested HNE cells can be used as surrogates for HBE cells in CFTR studies Reasonable availability/accessibility, particularly for rare CF genotypes Potential usage to investigate modulation of alternative channels/transporters or downstream consequences of CFTR dysfunction Results from HNE cultures correlate well with in vivo biomarkers/clinical features	Low throughput/limited scalability to assess multiple compounds (or combinations thereof) Lack of standardized procedures
Airway organoids	Cultures achieve maturity and readiness for usage faster than ALI cultures Greater throughput/scalability than ALI cultures to assess multiple compounds	Requirement of invasive procedures to be harvested if derived from HBE cells Greater variability in results compared to ALI cultures Unfavorable response-to-background ratio Lack of standardized procedures
Rectal biopsies	Abundant expression of CFTR in distal colon tissue ICM is a sensitive biomarker of CFTR function	Requirement of invasive procedures (although with no to low pain associated) Requirement of testing all samples on the harvesting day (i.e., cannot be biobanked) Possible reduced penetration of CFTR modulators under ex vivo conditions
Intestinal organoids	High throughput/scalability to assess multiple compounds (or combinations thereof) Reasonable availability/accessibility, particularly for rare CF genotypes Results from FIS assay correlate well with in vivo biomarkers/clinical features	Requirement of invasive procedures, since they are derived from rectal biopsies Unclear potential to investigate modulation of alternative channels/transporters

## References

[1] Ratjen F., Bell S.C., Rowe S.M., Goss C.H., Quittner A.L., Bush A. (2015). Cystic fibrosis. Nat. Rev. Dis. Prim..

[2] Lopes-Pacheco M. (2020). CFTR Modulators: The Changing Face of Cystic Fibrosis in the Era of Precision Medicine. Front. Pharmacol..

[3] Rommens J.M., Iannuzzi M.C., Kerem B., Drumm M.L., Melmer G., Dean M., Rozmahel R., Cole J.L., Kennedy D., Hidaka N. (1989). Identification of the cystic fibrosis gene: Chromosome walking and jumping. Science.

[4] Riordan J.R., Rommens J.M., Kerem B.S., Alon N.O.A., Rozmahel R., Grzelczak Z., Zielenski J., Lok S.I., Plavsic N., Chou J.L. (1989). Identification of the Cystic Fibrosis Gene: Cloning and Characterization of Complementary DNA. Science.

[5] Kerem B., Rommens J.M., Buchanan J.A., Markiewicz D., Cox T.K., Chakravarti A., Buchwald M., Tsui L. (1989). Identification of the cystic fibrosis gene: Genetic analysis. Science.

[6] Lopes-Pacheco M. (2016). CFTR Modulators: Shedding Light on Precision Medicine for Cystic Fibrosis. Front. Pharmacol..

[7] Cohen-Cymberknoh M., Shoseyov D., Kerem E. (2011). Managing Cystic Fibrosis: Strategies that increase life expectancy and improve quality of life. Am. J. Respir. Crit. Care Med..

[8] Castellani C., Duff A.J.A., Bell S.C., Heijerman H.G.M., Munck A., Ratjen F., Sermet-Gaudelus I., Southern K.W., Barben J., Flume P.A. (2018). ECFS best practice guidelines: The 2018 revision. J. Cyst. Fibros..

[9] Bell S.C., Mall M.A., Gutierrez H., Macek M., Madge S., Davies J.C., Burgel P.R., Tullis E., Castaños C., Castellani C. (2020). The future of cystic fibrosis care: A global perspective. Lancet Respir. Med..

[10] Lopes-Pacheco M., Pedemonte N., Kicic A. (2019). Editorial: Emerging Therapeutic Approaches for Cystic Fibrosis. Front. Pharmacol..

[11] Narayanan S., Mainz J.G., Gala S., Tabori H., Grossoehme D. (2017). Adherence to therapies in cystic fibrosis: A targeted literature review. Expert Rev. Respir. Med..

[12] Pinto M.C., Silva I.A.L., Figueira M.F., Amaral M.D., Lopes-Pacheco M. (2021). Pharmacological Modulation of Ion Channels for the Treatment of Cystic Fibrosis. J. Exp. Pharmacol..

[13] Lopes-Pacheco M., Pedemonte N., Veit G. (2021). Discovery of CFTR modulators for the treatment of cystic fibrosis. Expert Opin. Drug Discov..

[14] Gruenert D.C., Willems M., Cassiman J.J., Frizzell R.A. (2004). Established cell lines used in cystic fibrosis research. J. Cyst. Fibros..

[15] Clancy J.P., Cotton C.U., Donaldson S.H., Solomon G.M., VanDevanter D.R., Boyle M.P., Gentzsch M., Nick J.A., Illek B., Wallenburg J.C. (2019). CFTR modulator theratyping: Current status, gaps and future directions. J. Cyst. Fibros..

[16] Pedemonte N., Lukacs G.L., Du K., Caci E., Zegarra-Moran O., Galietta L.J.V., Verkman A.S. (2005). Small-molecule correctors of defective ΔF508-CFTR cellular processing identified by high-throughput screening. J. Clin. Investig..

[17] Van Goor F., Straley K.S., Cao D., González J., Hadida S., Hazlewood A., Joubran J., Knapp T., Makings L.R., Miller M. (2006). Rescue of ΔF508-CFTR trafficking and gating in human cystic fibrosis airway primary cultures by small molecules. Am. J. Physiol. Cell. Mol. Physiol..

[18] Robert R., Carlile G.W., Pavel C., Liu N., Anjos S.M., Liao J., Luo Y., Zhang D., Thomas D.Y., Hanrahan J.W. (2008). Structural Analog of Sildenafil Identified as a Novel Corrector of the F508del-CFTR Trafficking Defect. Mol. Pharmacol..

[19] Veit G., Xu H., Dreano E., Avramescu R.G., Bagdany M., Beitel L.K., Roldan A., Hancock M., Lay C., Li W. (2018). Structure-guided combination therapy to potently improve the function of mutant CFTRs. Nat. Med..

[20] Ramsey B.W., Davies J.C., McElvaney N.G., Tullis E., Bell S.C., Drevinek P., Griese M., McKone E.F., Wainwright C.E., Konstan M.W. (2011). A CFTR potentiator in patients with cystic fibrosis and the G551D mutation. N. Engl. J. Med..

[21] McKone E.F., Borowitz D., Drevinek P., Griese M., Konstan M.W., Wainwright C., Ratjen F., Sermet-Gaudelus I., Plant B., Munck A. (2014). Long-term safety and efficacy of ivacaftor in patients with cystic fibrosis who have the Gly551Asp- CFTR mutation: A phase 3, open-label extension study (PERSIST). Lancet Respir. Med..

[22] Clancy J.P., Rowe S.M., Accurso F.J., Aitken M.L., Amin R.S., Ashlock M.A., Ballmann M., Boyle M.P., Bronsveld I., Campbell P.W. (2012). Results of a phase IIa study of VX-809, an investigational CFTR corrector compound, in subjects with cystic fibrosis homozygous for the*F508del-CFTR* mutation. Thorax.

[23] Flume P.A., Liou T.G., Borowitz D.S., Li H., Yen K., Ordoñez C.L., Geller D.E. (2012). Ivacaftor in Subjects with Cystic Fibrosis Who Are Homozygous for the F508del-CFTR Mutation. Chest.

[24] Wainwright C.E., Elborn J.S., Ramsey B.W., Marigowda G., Huang X., Cipolli M., Colombo C., Davies J.C., De Boeck K., Flume P.A. (2015). Lumacaftor–Ivacaftor in Patients with Cystic Fibrosis Homozygous for Phe508del CFTR. N. Engl. J. Med..

[25] Konstan M.W., McKone E.F., Moss R.B., Marigowda G., Tian S., Waltz D., Huang X., Lubarsky B., Rubin J., Millar S.J. (2017). Assessment of safety and efficacy of long-term treatment with combination lumacaftor and ivacaftor therapy in patients with cystic fibrosis homozygous for the F508del-CFTR mutation (PROGRESS): A phase 3, extension study. Lancet Respir. Med..

[26] Taylor-Cousar J.L., Munck A., McKone E.F., van der Ent C.K., Moeller A., Simard C., Wang L.T., Ingenito E.P., McKee C., Lu Y. (2017). Tezacaftor–Ivacaftor in Patients with Cystic Fibrosis Homozygous for Phe508del. N. Engl. J. Med..

[27] Rowe S.M., Daines C., Ringshausen F.C., Kerem E., Wilson J., Tullis E., Nair N., Simard C., Han L., Ingenito E.P. (2017). Tezacaftor–Ivacaftor in Residual-Function Heterozygotes with Cystic Fibrosis. N. Engl. J. Med..

[28] Heijerman H.G.M., McKone E.F., Downey D.G., Van Braeckel E., Rowe S.M., Tullis E., Mall M.A., Welter J.J., Ramsey B.W., McKee C.M. (2019). Efficacy and safety of the elexacaftor plus tezacaftor plus ivacaftor combination regimen in people with cystic fibrosis homozygous for the F508del mutation: A double-blind, randomised, phase 3 trial. Lancet.

[29] Middleton P.G., Mall M.A., Dřevínek P., Lands L.C., McKone E.F., Polineni D., Ramsey B.W., Taylor-Cousar J.L., Tullis E., Vermeulen F. (2019). Elexacaftor–Tezacaftor–Ivacaftor for Cystic Fibrosis with a Single Phe508del Allele. N. Engl. J. Med..

[30] Costa E., Girotti S., Pauro F., Leufkens H.G.M., Cipolli M. (2022). The impact of FDA and EMA regulatory decision-making process on the access to CFTR modulators for the treatment of cystic fibrosis. Orphanet J. Rare Dis..

[31] Farinha C.M., Sousa M., Canato S., Schmidt A., Uliyakina I., Amaral M.D. (2015). Increased efficacy of VX -809 in different cellular systems results from an early stabilization effect of F508del-CFTR. Pharmacol. Res. Perspect..

[32] Avramescu R.G., Kai Y., Xu H., Bidaud-Meynard A., Schnúr A., Frenkiel S., Matouk E., Veit G., Lukacs G.L. (2017). Mutation-specific downregulation of CFTR2 variants by gating potentiators. Hum. Mol. Genet..

[33] Mogayzel P.J., Henning K.A., Bittner M.L., Novotny E.A., Schwiebert E.M., Guggino W.B., Jiang Y., Rosenfeld M.A. (1997). Functional human CFTR produced by stable Chinese hamster ovary cell lines derived using yeast artificial chromosomes. Hum. Mol. Genet..

[34] Weber A.J., Soong G., Bryan R., Saba S., Prince A. (2001). Activation of NF-κB in airway epithelial cells is dependent on CFTR trafficking and Cl^−^ channel function. Am. J. Physiol. Cell. Mol. Physiol..

[35] Bose S.J., Bijvelds M.J.C., Wang Y., Liu J., Cai Z., Bot A.G.M., de Jonge H.R., Sheppard D.N. (2019). Differential thermostability and response to cystic fibrosis transmembrane conductance regulator potentiators of human and mouse F508del-CFTR. Am. J. Physiol. Cell. Mol. Physiol..

[36] Seibert F.S., Linsdell P., Loo T.W., Hanrahan J.W., Riordan J.R., Clarke D.M. (1996). Cytoplasmic Loop Three of Cystic Fibrosis Transmembrane Conductance Regulator Contributes to Regulation of Chloride Channel Activity. J. Biol. Chem..

[37] Rapino D., Sabirzhanova I., Lopes-Pacheco M., Grover R., Guggino W.B., Cebotaru L. (2015). Rescue of NBD2 Mutants N1303K and S1235R of CFTR by Small-Molecule Correctors and Transcomplementation. PLoS ONE.

[38] Lopes-Pacheco M., Sabirzhanova I., Rapino D., Morales M.M., Guggino W.B., Cebotaru L. (2016). Correctors Rescue CFTR Mutations in Nucleotide-Binding Domain 1 (NBD1) by Modulating Proteostasis. ChemBioChem.

[39] Lopes-Pacheco M., Boinot C., Sabirzhanova I., Rapino D., Cebotaru L.L. (2017). Combination of Correctors Rescues CFTR Transmembrane-Domain Mutants by Mitigating their Interactions with Proteostasis. Cell. Physiol. Biochem..

[40] Caputo A., Hinzpeter A., Caci E., Pedemonte N., Arous N., Di Duca M., Zegarra-Moran O., Fanen P., Galietta L.J.V. (2009). Mutation-Specific Potency and Efficacy of Cystic Fibrosis Transmembrane Conductance Regulator Chloride Channel Potentiators. J. Pharmacol. Exp. Ther..

[41] Lee M.G., Wigley W.C., Zeng W., Noel L.E., Marino C.R., Thomas P.J., Muallem S. (1999). Regulation of Cl−/HCO3−Exchange by Cystic Fibrosis Transmembrane Conductance Regulator Expressed in NIH 3T3 and HEK 293 Cells. J. Biol. Chem..

[42] Lopes-Pacheco M., Boinot C., Sabirzhanova I., Morales M.M., Guggino W.B., Cebotaru L. (2015). Combination of Correctors Rescue ΔF508-CFTR by Reducing Its Association with Hsp40 and Hsp27. J. Biol. Chem..

[43] Sharma N., Evans T.A., Pellicore M.J., Davis E., Aksit M.A., McCague A.F., Joynt A.T., Lu Z., Han S.T., Anzmann A.F. (2018). Capitalizing on the heterogeneous effects of CFTR nonsense and frameshift variants to inform therapeutic strategy for cystic fibrosis. PLoS Genet..

[44] Patel W., Moore P.J., Sassano M.F., Lopes-Pacheco M., Aleksandrov A.A., Amaral M.D., Tarran R., Gray M.A. (2019). Increases in cytosolic Ca2+ induce dynamin- and calcineurin-dependent internalisation of CFTR. Cell. Mol. Life Sci..

[45] Moyer B.D., Loffing J., Schwiebert E.M., Loffing-Cueni D., Halpin P.A., Karlson K.H., Ismailov I.I., Guggino W.B., Langford G.M., Stanton B.A. (1998). Membrane Trafficking of the Cystic Fibrosis Gene Product, Cystic Fibrosis Transmembrane Conductance Regulator, Tagged with Green Fluorescent Protein in Madin-Darby Canine Kidney Cells. J. Biol. Chem..

[46] Krasnov K.V., Tzetis M., Cheng J., Guggino W.B., Cutting G.R. (2008). Localization studies of rare missense mutations in cystic fibrosis transmembrane conductance regulator (CFTR) facilitate interpretation of genotype-phenotype relationships. Hum. Mutat..

[47] Van Goor F., Yu H., Burton B., Hoffman B.J. (2014). Effect of ivacaftor on CFTR forms with missense mutations associated with defects in protein processing or function. J. Cyst. Fibros..

[48] Lopes-Pacheco M., Silva I.A.L., Turner M.J., Carlile G.W., Sondo E., Thomas D.Y., Pedemonte N., Hanrahan J.W., Amaral M.D. (2020). Characterization of the mechanism of action of RDR01752, a novel corrector of F508del-CFTR. Biochem. Pharmacol..

[49] Han S.T., Rab A., Pellicore M.J., Davis E.F., McCague A.F., Evans T.A., Joynt A.T., Lu Z., Cai Z., Raraigh K.S. (2018). Residual function of cystic fibrosis mutants predicts response to small molecule CFTR modulators. JCI Insight.

[50] Cozens A.L., Yezzi M.J., Kunzelmann K., Ohrui T., Chin L., Eng K., Finkbeiner W.E., Widdicombe J.H., Gruenert D.C. (1994). CFTR expression and chloride secretion in polarized immortal human bronchial epithelial cells. Am. J. Respir. Cell Mol. Biol..

[51] Bebok Z., Collawn J.F., Wakefield J., Parker W., Li Y., Varga K., Sorscher E.J., Clancy J.P. (2005). Failure of cAMP agonists to activate rescued ΔF508 CFTR in CFBE41o^−^ airway epithelial monolayers. J. Physiol..

[52] Lopes-Pacheco M., Bacalhau M., Ramalho S.S., Silva I.A.L., Ferreira F.C., Carlile G.W., Thomas D.Y., Farinha C.M., Hanrahan J.W., Amaral M.D. (2022). Rescue of Mutant CFTR Trafficking Defect by the Investigational Compound MCG1516A. Cells.

[53] Yu H., Burton B., Huang C.J., Worley J., Cao D., Johnson J.P., Urrutia A., Joubran J., Seepersaud S., Sussky K. (2012). Ivacaftor potentiation of multiple CFTR channels with gating mutations. J. Cyst. Fibros..

[54] Amato F., Scudieri P., Musante I., Tomati V., Caci E., Comegna M., Maietta S., Manzoni F., Di Lullo A.M., De Wachter E. (2019). Two CFTR mutations within codon 970 differently impact on the chloride channel functionality. Hum. Mutat..

[55] Fidler M.C., Buckley A., Sullivan J.C., Statia M., Boj S.F., Vries R.G.J., Munck A., Higgins M., Moretto Zita M., Negulescu P. (2021). *G970R-CFTR* Mutation (c.2908G>C) Results Predominantly in a Splicing Defect. Clin. Transl. Sci..

[56] De Boeck K., Munck A., Walker S., Faro A., Hiatt P., Gilmartin G., Higgins M. (2014). Efficacy and safety of ivacaftor in patients with cystic fibrosis and a non-G551D gating mutation. J. Cyst. Fibros..

[57] Oren Y.S., Irony-Tur Sinai M., Golec A., Barchad-Avitzur O., Mutyam V., Li Y., Hong J., Ozeri-Galai E., Hatton A., Leibson C. (2021). Antisense oligonucleotide-based drug development for Cystic Fibrosis patients carrying the 3849 + 10 kb C-to-T splicing mutation. J. Cyst. Fibros..

[58] Nissim-Rafinia M., Aviram M., Randell S.H., Shushi L., Ozeri E., Chiba-Falek O., Eidelman O., Pollard H.B., Yankaskas J.R., Kerem B. (2004). Restoration of the cystic fibrosis transmembrane conductance regulator function by splicing modulation. EMBO Rep..

[59] Joynt A.T., Evans T.A., Pellicore M.J., Davis-Marcisak E.F., Aksit M.A., Eastman A.C., Patel S.U., Paul K.C., Osorio D.L., Bowling A.D. (2020). Evaluation of both exonic and intronic variants for effects on RNA splicing allows for accurate assessment of the effectiveness of precision therapies. PLoS Genet..

[60] Ehrhardt C., Collnot E.M., Baldes C., Becker U., Laue M., Kim K.J., Lehr C.M. (2006). Towards an in vitro model of cystic fibrosis small airway epithelium: Characterisation of the human bronchial epithelial cell line CFBE41o^−^. Cell Tissue Res..

[61] Lundberg A.S., Randell S.H., Stewart S.A., Elenbaas B., Hartwell K.A., Brooks M.W., Fleming M.D., Olsen J.C., Miller S.W., Weinberg R.A. (2002). Immortalization and transformation of primary human airway epithelial cells by gene transfer. Oncogene.

[62] Pedemonte N., Tomati V., Sondo E., Galietta L.J.V. (2010). Influence of cell background on pharmacological rescue of mutant CFTR. Am. J. Physiol. Physiol..

[63] Sondo E., Tomati V., Caci E., Esposito A.I., Pfeffer U., Pedemonte N., Galietta L.J.V. (2011). Rescue of the mutant CFTR chloride channel by pharmacological correctors and low temperature analyzed by gene expression profiling. Am. J. Physiol. Physiol..

[64] Ostedgaard L.S., Rogers C.S., Dong Q., Randak C.O., Vermeer D.W., Rokhlina T., Karp P.H., Welsh M.J. (2007). Processing and function of CFTR-ΔF508 are species-dependent. Proc. Natl. Acad. Sci. USA.

[65] Gottschalk L.B., Vecchio-Pagan B., Sharma N., Han S.T., Franca A., Wohler E.S., Batista D.A.S., Goff L.A., Cutting G.R. (2015). Creation and characterization of an airway epithelial cell line for stable expression of CFTR variants. J. Cyst. Fibros..

[66] Ehrhardt C., Kneuer C., Fiegel J., Hanes J., Schaefer U., Kim K.J., Lehr C.M. (2002). Influence of apical fluid volume on the development of functional intercellular junctions in the human epithelial cell line 16HBE14o-: Implications for the use of this cell line as an in vitro model for bronchial drug absorption studies. Cell Tissue Res..

[67] Valley H.C., Bukis K.M., Bell A., Cheng Y., Wong E., Jordan N.J., Allaire N.E., Sivachenko A., Liang F., Bihler H. (2019). Isogenic cell models of cystic fibrosis-causing variants in natively expressing pulmonary epithelial cells. J. Cyst. Fibros..

[68] Venturini A., Borrelli A., Musante I., Scudieri P., Capurro V., Renda M., Pedemonte N., Galietta L.J.V. (2021). Comprehensive Analysis of Combinatorial Pharmacological Treatments to Correct Nonsense Mutations in the CFTR Gene. Int. J. Mol. Sci..

[69] Laselva O., Bartlett C., Popa A., Ouyang H., Gunawardena T.N.A., Gonska T., Moraes T.J., Bear C.E. (2021). Emerging preclinical modulators developed for F508del-CFTR have the potential to be effective for ORKAMBI resistant processing mutants. J. Cyst. Fibros..

[70] Laselva O., McCormack J., Bartlett C., Ip W., Gunawardena T.N.A., Ouyang H., Eckford P.D.W., Gonska T., Moraes T.J., Bear C.E. (2020). Preclinical Studies of a Rare CFCausing Mutation in the Second Nucleotide Binding Domain (c.3700A>G) Show Robust Functional Rescue in Primary Nasal Cultures by Novel CFTR Modulators. J. Pers. Med..

[71] Phuan P.W., Haggie P.M., Tan J.A., Rivera A.A., Finkbeiner W.E., Nielson D.W., Thomas M.M., Janahi I.A., Verkman A.S. (2021). CFTR modulator therapy for cystic fibrosis caused by the rare c.3700A>G mutation. J. Cyst. Fibros..

[72] Santos L., Mention K., Cavusoglu-Doran K., Sanz D.J., Bacalhau M., Lopes-Pacheco M., Harrison P.T., Farinha C.M. (2022). Comparison of Cas9 and Cas12a CRISPR editing methods to correct the W1282XCFTR mutation. J. Cyst. Fibros..

[73] Jiang T., Henderson J.M., Coote K., Cheng Y., Valley H.C., Zhang X.O., Wang Q., Rhym L.H., Cao Y., Newby G.A. (2020). Chemical modifications of adenine base editor mRNA and guide RNA expand its application scope. Nat. Commun..

[74] Guimbellot J., Solomon G.M., Baines A., Heltshe S.L., VanDalfsen J., Joseloff E., Sagel S.D., Rowe S.M. (2019). Effectiveness of ivacaftor in cystic fibrosis patients with non-G551D gating mutations. J. Cyst. Fibros..

[75] Salvatore D., Carnovale V., Iacotucci P., Braggion C., Castellani C., Cimino G., Colangelo C., Francalanci M., Leonetti G., Lucidi V. (2019). Effectivenesss of ivacaftor in severe cystic fibrosis patients and non-G551D gating mutations. Pediatr. Pulmonol..

[76] McCague A.F., Raraigh K.S., Pellicore M.J., Davis-Marcisak E.F., Evans T.A., Han S.T., Lu Z., Joynt A.T., Sharma N., Castellani C. (2019). Correlating Cystic Fibrosis Transmembrane Conductance Regulator Function with Clinical Features to Inform Precision Treatment of Cystic Fibrosis. Am. J. Respir. Crit. Care Med..

[77] Dekkers J.F., Berkers G., Kruisselbrink E., Vonk A., de Jonge H.R., Janssens H.M., Bronsveld I., van de Graaf E.A., Nieuwenhuis E.E.S., Houwen R.H.J. (2016). Characterizing responses to CFTR-modulating drugs using rectal organoids derived from subjects with cystic fibrosis. Sci. Transl. Med..

[78] Berkers G., van Mourik P., Vonk A.M., Kruisselbrink E., Dekkers J.F., de Winter-de Groot K.M., Arets H.G.M., Marck-van der Wilt R.E.P., Dijkema J.S., Vanderschuren M.M. (2019). Rectal Organoids Enable Personalized Treatment of Cystic Fibrosis. Cell Rep..

[79] Pranke I.M., Hatton A., Simonin J., Jais J.P., Le Pimpec-Barthes F., Carsin A., Bonnette P., Fayon M., Stremler-Le Bel N., Grenet D. (2017). Correction of CFTR function in nasal epithelial cells from cystic fibrosis patients predicts improvement of respiratory function by CFTR modulators. Sci. Rep..

[80] Donaldson S.H., Pilewski J.M., Griese M., Cooke J., Viswanathan L., Tullis E., Davies J.C., Lekstrom-Himes J.A., Wang L.T. (2018). Tezacaftor/Ivacaftor in Subjects with Cystic Fibrosis and F508del/F508del-CFTR or F508del/G551DCFTR. Am. J. Respir. Crit. Care Med..

[81] Sepahzad A., Morris-Rosendahl D.J., Davies J.C. (2021). Cystic Fibrosis Lung Disease Modifiers and Their Relevance in the New Era of Precision Medicine. Genes.

[82] McGarry M.E., McColley S.A. (2021). Cystic fibrosis patients of minority race and ethnicity less likely eligible for CFTR modulators based on *CFTR* genotype. Pediatr. Pulmonol..

[83] Van Goor F., Hadida S., Grootenhuis P.D.J., Burton B., Cao D., Neuberger T., Turnbull A., Singh A., Joubran J., Hazlewood A. (2009). Rescue of CF airway epithelial cell function in vitro by a CFTR potentiator, VX-770. Proc. Natl. Acad. Sci. USA.

[84] Van Goor F., Hadida S., Grootenhuis P.D.J., Burton B., Stack J.H., Straley K.S., Decker C.J., Miller M., McCartney J., Olson E.R. (2011). Correction of the F508del-CFTR protein processing defect in vitro by the investigational drug VX-809. Proc. Natl. Acad. Sci. USA.

[85] Fulcher M.L., Randell S.H. (2013). Human nasal and tracheo-bronchial respiratory epithelial cell culture. Methods Mol. Biol..

[86] Liu X., Ory V., Chapman S., Yuan H., Albanese C., Kallakury B., Timofeeva O.A., Nealon C., Dakic A., Simic V. (2012). ROCK Inhibitor and Feeder Cells Induce the Conditional Reprogramming of Epithelial Cells. Am. J. Pathol..

[87] Liu X., Krawczyk E., Suprynowicz F.A., Palechor-Ceron N., Yuan H., Dakic A., Simic V., Zheng Y.L., Sripadhan P., Chen C. (2017). Conditional reprogramming and long-term expansion of normal and tumor cells from human biospecimens. Nat. Protoc..

[88] Mou H., Vinarsky V., Tata P.R., Brazauskas K., Choi S.H., Crooke A.K., Zhang B., Solomon G.M., Turner B., Bihler H. (2016). Dual SMAD Signaling Inhibition Enables Long-Term Expansion of Diverse Epithelial Basal Cells. Cell Stem Cell.

[89] Gentzsch M., Boyles S.E., Cheluvaraju C., Chaudhry I.G., Quinney N.L., Cho C., Dang H., Liu X., Schlegel R., Randell S.H. (2017). Pharmacological Rescue of Conditionally Reprogrammed Cystic Fibrosis Bronchial Epithelial Cells. Am. J. Respir. Cell Mol. Biol..

[90] Wu X., Wang S., Li M., Li J., Shen J., Zhao Y., Pang J., Wen Q., Chen M., Wei B. (2020). Conditional reprogramming: Next generation cell culture. Acta Pharm. Sin. B.

[91] Itani O.A., Chen J.H., Karp P.H., Ernst S., Keshavjee S., Parekh K., Klesney-Tait J., Zabner J., Welsh M.J. (2011). Human cystic fibrosis airway epithelia have reduced Cl^−^ conductance but not increased Na^+^ conductance. Proc. Natl. Acad. Sci. USA.

[92] Coakley R.D., Grubb B.R., Paradiso A.M., Gatzy J.T., Johnson L.G., Kreda S.M., O’Neal W.K., Boucher R.C. (2003). Abnormal surface liquid pH regulation by cultured cystic fibrosis bronchial epithelium. Proc. Natl. Acad. Sci. USA.

[93] Birket S.E., Chu K.K., Liu L., Houser G.H., Diephuis B.J., Wilsterman E.J., Dierksen G., Mazur M., Shastry S., Li Y. (2014). A Functional Anatomic Defect of the Cystic Fibrosis Airway. Am. J. Respir. Crit. Care Med..

[94] Wu Y.S., Jiang J., Ahmadi S., Lew A., Laselva O., Xia S., Bartlett C., Ip W., Wellhauser L., Ouyang H. (2019). ORKAMBIMediated Rescue of Mucociliary Clearance in Cystic Fibrosis Primary Respiratory Cultures Is Enhanced by Arginine Uptake, Arginase Inhibition, and Promotion of Nitric Oxide Signaling to the Cystic Fibrosis Transmembrane Conductance Regulator Channel. Mol. Pharmacol..

[95] Brewington J.J., Filbrandt E.T., LaRosa F.J., Moncivaiz J.D., Ostmann A.J., Strecker L.M., Clancy J.P. (2018). Brushed nasal epithelial cells are a surrogate for bronchial epithelial CFTR studies. JCI Insight.

[96] Müller L., Brighton L.E., Carson J.L., Fischer W.A., Jaspers I. (2013). Culturing of Human Nasal Epithelial Cells at the Air Liquid Interface. J. Vis. Exp..

[97] Sette G., Cicero S.L., Blaconà G., Pierandrei S., Bruno S.M., Salvati V., Castelli G., Falchi M., Fabrizzi B., Cimino G. (2021). Theratyping cystic fibrosis in vitro in ALI culture and organoid models generated from patient-derived nasal epithelial conditionally reprogrammed stem cells. Eur. Respir. J..

[98] Noel S., Servel N., Hatton A., Golec A., Rodrat M., Ng D.R.S., Li H., Pranke I., Hinzpeter A., Edelman A. (2022). Correlating genotype with phenotype using CFTR-mediated whole-cell Cl ^−^ currents in human nasal epithelial cells. J. Physiol..

[99] Montoro D.T., Haber A.L., Biton M., Vinarsky V., Lin B., Birket S.E., Yuan F., Chen S., Leung H.M., Villoria J. (2018). A revised airway epithelial hierarchy includes CFTR-expressing ionocytes. Nature.

[100] Plasschaert L.W., Žilionis R., Choo-Wing R., Savova V., Knehr J., Roma G., Klein A.M., Jaffe A.B. (2018). A single-cell atlas of the airway epithelium reveals the CFTR-rich pulmonary ionocyte. Nature.

[101] Scudieri P., Musante I., Venturini A., Guidone D., Genovese M., Cresta F., Caci E., Palleschi A., Poeta M., Santamaria F. (2020). Ionocytes and CFTR Chloride Channel Expression in Normal and Cystic Fibrosis Nasal and Bronchial Epithelial Cells. Cells.

[102] Okuda K., Dang H., Kobayashi Y., Carraro G., Nakano S., Chen G., Kato T., Asakura T., Gilmore R.C., Morton L.C. (2021). Secretory Cells Dominate Airway CFTR Expression and Function in Human Airway Superficial Epithelia. Am. J. Respir. Crit. Care Med..

[103] Pranke I., Hatton A., Masson A., Flament T., Le Bourgeois M., Chedevergne F., Bailly C., Urbach V., Hinzpeter A., Edelman A. (2019). Might Brushed Nasal Cells Be a Surrogate for CFTR Modulator Clinical Response?. Am. J. Respir. Crit. Care Med..

[104] McDougall C.M., Blaylock M.G., Douglas J.G., Brooker R.J., Helms P.J., Walsh G.M. (2008). Nasal Epithelial Cells as Surrogates for Bronchial Epithelial Cells in Airway Inflammation Studies. Am. J. Respir. Cell Mol. Biol..

[105] de Courcey F., Zholos A.V., Atherton-Watson H., Williams M.T.S., Canning P., Danahay H.L., Elborn J.S., Ennis M. (2012). Development of primary human nasal epithelial cell cultures for the study of cystic fibrosis pathophysiology. Am. J. Physiol. Physiol..

[106] Anderson J.D., Liu Z., Odom L.V., Kersh L., Guimbellot J.S. (2021). CFTR function and clinical response to modulators parallel nasal epithelial organoid swelling. Am. J. Physiol. Cell. Mol. Physiol..

[107] Zabner J., Couture L.A., Gregory R.J., Graham S.M., Smith A.E., Welsh M. (1993). Adenovirus-mediated gene transfer transiently corrects the chloride transport defect in nasal epithelia of patients with cystic fibrosis. Cell.

[108] Cao H., Ouyang H., Laselva O., Bartlett C., Zhou Z.P., Duan C., Gunawardena T., Avolio J., Bear C.E., Gonska T. (2020). A helper-dependent adenoviral vector rescues CFTR to wild-type functional levels in cystic fibrosis epithelial cells harbouring class I mutations. Eur. Respir. J..

[109] Oren Y.S., Avizur-Barchad O., Ozeri-Galai E., Elgrabli R., Schirelman M.R., Blinder T., Stampfer C.D., Ordan M., Laselva O., Cohen-Cymberknoh M. (2021). Antisense oligonucleotide splicing modulation as a novel Cystic Fibrosis therapeutic approach for the W1282X nonsense mutation. J. Cyst. Fibros..

[110] Lopes-Pacheco M., Kitoko J.Z., Morales M.M., Petrs-Silva H., Rocco P.R.M. (2018). Self-complementary and tyrosine-mutant rAAV vectors enhance transduction in cystic fibrosis bronchial epithelial cells. Exp. Cell Res..

[111] Roomans G.M., Kozlova I., Nilsson H., Vanthanouvong V., Button B., Tarran R. (2004). Measurements of airway surface liquid height and mucus transport by fluorescence microscopy, and of ion composition by X-ray microanalysis. J. Cyst. Fibros..

[112] Cho D.Y., Hwang P.H., Illek B., Fischer H. (2011). Acid and base secretion in freshly excised nasal tissue from cystic fibrosis patients with ΔF508 mutation. Int. Forum Allergy Rhinol..

[113] Guimbellot J.S., Leach J.M., Chaudhry I.G., Quinney N.L., Boyles S.E., Chua M., Aban I., Jaspers I., Gentzsch M. (2017). Nasospheroids permit measurements of CFTR-dependent fluid transport. JCI Insight.

[114] Brewington J.J., Filbrandt E.T., LaRosa F.J., Ostmann A.J., Strecker L.M., Szczesniak R.D., Clancy J.P. (2018). Detection of CFTR function and modulation in primary human nasal cell spheroids. J. Cyst. Fibros..

[115] Debley J.S., Barrow K.A., Rich L.M., Singh P., McKone E.F., Nichols D.P. (2020). Correlation between Ivacaftor-induced CFTR Activation in Airway Epithelial Cells and Improved Lung Function: A Proof-of-Concept Study. Ann. Am. Thorac. Soc..

[116] McGarry M.E., Illek B., Ly N.P., Zlock L., Olshansky S., Moreno C., Finkbeiner W.E., Nielson D.W. (2017). In vivo and in vitro ivacaftor response in cystic fibrosis patients with residual CFTR function: N-of-1 studies. Pediatr. Pulmonol..

[117] Park J.K.H., Shrivastava A., Zhang C., Pollok B.A., Finkbeiner W.E., Gibb E.R., Ly N.P., Illek B. (2020). Functional Profiling of CFTRDirected Therapeutics Using Pediatric Patient-Derived Nasal Epithelial Cell Models. Front. Pediatr..

[118] Sachs N., Papaspyropoulos A., Zomer-van Ommen D.D., Heo I., Böttinger L., Klay D., Weeber F., Huelsz-Prince G., Iakobachvili N., Amatngalim G.D. (2019). Long-term expanding human airway organoids for disease modeling. EMBO J..

[119] Calucho M., Gartner S., Barranco P., Fernández-Álvarez P., Pérez R.G., Tizzano E.F. (2021). Validation of nasospheroids to assay CFTR functionality and modulator responses in cystic fibrosis. Sci. Rep..

[120] Li Y., Wu Q., Sun X., Shen J., Chen H. (2020). Organoids as a Powerful Model for Respiratory Diseases. Stem Cells Int..

[121] Cidem A., Bradbury P., Traini D., Ong H.X. (2020). Modifying and Integrating in vitro and ex vivo Respiratory Models for Inhalation Drug Screening. Front. Bioeng. Biotechnol..

[122] Tan C.L., Chan Y., Candasamy M., Chellian J., Madheswaran T., Sakthivel L.P., Patel V.K., Chakraborty A., MacLoughlin R., Kumar D. (2022). Unravelling the molecular mechanisms underlying chronic respiratory diseases for the development of novel therapeutics via in vitro experimental models. Eur. J. Pharmacol..

[123] Liu Z., Anderson J.D., Deng L., Mackay S., Bailey J., Kersh L., Rowe S.M., Guimbellot J.S. (2020). Human Nasal Epithelial Organoids for Therapeutic Development in Cystic Fibrosis. Genes.

[124] Mall M., Bleich M., Schürlein M., Kühr J., Seydewitz H.H., Brandis M., Greger R., Kunzelmann K. (1998). Cholinergic ion secretion in human colon requires coactivation by cAMP. Am. J. Physiol. Content.

[125] Kunzelmann K., Mall M. (2002). Electrolyte Transport in the Mammalian Colon: Mechanisms and Implications for Disease. Physiol. Rev..

[126] Mall M., Wissner A., Seydewitz H.H., Kuehr J., Brandis M., Greger R., Kunzelmann K. (2000). Defective cholinergic Cl^−^ secretion and detection of K^+^ secretion in rectal biopsies from cystic fibrosis patients. Am. J. Physiol. Liver Physiol..

[127] Veeze H.J., Sinaasappel M., Bijman J., Bouquet J., De Jonge H.R. (1991). Ion transport abnormalities in rectal suction biopsies from children with cystic fibrosis. Gastroenterology.

[128] Graeber S.Y., Vitzthum C., Mall M.A. (2021). Potential of Intestinal Current Measurement for Personalized Treatment of Patients with Cystic Fibrosis. J. Pers. Med..

[129] Veeze H.J., Halley D.J.J., Bijman J., de Jongste J.C., de Jonge H.R., Sinaasappel M. (1994). Determinants of mild clinical symptoms in cystic fibrosis patients. Residual chloride secretion measured in rectal biopsies in relation to the genotype. J. Clin. Investig..

[130] Derichs N., Sanz J., Von Kanel T., Stolpe C., Zapf A., Tümmler B., Gallati S., Ballmann M. (2010). Intestinal current measurement for diagnostic classification of patients with questionable cystic fibrosis: Validation and reference data. Thorax.

[131] de Jonge H.R., Ballmann M., Veeze H., Bronsveld I., Stanke F., Tümmler B., Sinaasappel M. (2004). Ex vivo CF diagnosis by intestinal current measurements (ICM) in small aperture, circulating Ussing chambers. J. Cyst. Fibros..

[132] Mall M., Hirtz S., Gonska T., Kunzelmann K. (2004). Assessment of CFTR function in rectal biopsies for the diagnosis of cystic fibrosis. J. Cyst. Fibros..

[133] Silva I.A.L., Duarte A., Marson F.A.L., Centeio R., Doušová T., Kunzelmann K., Amaral M.D. (2020). Assessment of Distinct Electrophysiological Parameters in Rectal Biopsies for the Choice of the Best Diagnosis/Prognosis Biomarkers for Cystic Fibrosis. Front. Physiol..

[134] Hirtz S., Gonska T., Seydewitz H.H., Thomas J., Greiner P., Kuehr J., Brandis M., Eichler I., Rocha H., Lopes A.I. (2004). CFTR Cl− channel function in native human colon correlates with the genotype and phenotype in cystic fibrosis. Gastroenterology.

[135] Farrell P.M., White T.B., Ren C.L., Hempstead S.E., Accurso F., Derichs N., Howenstine M., McColley S.A., Rock M., Rosenfeld M. (2017). Diagnosis of Cystic Fibrosis: Consensus Guidelines from the Cystic Fibrosis Foundation. J. Pediatr..

[136] Graeber S.Y., Hug M.J., Sommerburg O., Hirtz S., Hentschel J., Heinzmann A., Dopfer C., Schulz A., Mainz J.G., Tümmler B. (2015). Intestinal Current Measurements Detect Activation of Mutant CFTR in Patients with Cystic Fibrosis with the G551D Mutation Treated with Ivacaftor. Am. J. Respir. Crit. Care Med..

[137] Graeber S.Y., Dopfer C., Naehrlich L., Gyulumyan L., Scheuermann H., Hirtz S., Wege S., Mairbäurl H., Dorda M., Hyde R. (2018). Effects of Lumacaftor–Ivacaftor Therapy on Cystic Fibrosis Transmembrane Conductance Regulator Function in Phe508del Homozygous Patients with Cystic Fibrosis. Am. J. Respir. Crit. Care Med..

[138] Masson A., Schneider-Futschik E.K., Baatallah N., Nguyen-Khoa T., Girodon E., Hatton A., Flament T., Le Bourgeois M., Chedevergne F., Bailly C. (2019). Predictive factors for lumacaftor/ivacaftor clinical response. J. Cyst. Fibros..

[139] Graeber S.Y., Vitzhum C., Pallenber S.T., Naehrlich L., Stahl M., Rohrbach A., Drescher M., Minso R., Ringshausen F.C., Rueckes-Nilges C. (2022). Effects of Elexacaftor/Tezacaftor/Ivacaftor Therapy on CFTR Function in Patients with Cystic Fibrosis and One or Two *F508del* Alleles. Am. J. Respir. Crit. Care Med..

[140] Wilschanski M., Yaakov Y., Omari I., Zaman M., Martin C.R., Cohen-Cymberknoh M., Shoseyov D., Kerem E., Dasilva D., Sheth S. (2016). Comparison of Nasal Potential Difference and Intestinal Current Measurements as Surrogate Markers for CFTR Function. J. Pediatr. Gastroenterol. Nutr..

[141] Dekkers J.F., Wiegerinck C.L., De Jonge H.R., Bronsveld I., Janssens H.M., De Winter-de Groot K.M., Brandsma A.M., de Jong N.W.M., Bijvelds M.J.C., Scholte B.J. (2013). A functional CFTR assay using primary cystic fibrosis intestinal organoids. Nat. Med..

[142] Vonk A.M., van Mourik P., Ramalho A.S., Silva I.A.L., Statia M., Kruisselbrink E., Suen S.W.F., Dekkers J.F., Vleggaar F.P., Houwen R.H.J. (2020). Protocol for Application, Standardization and Validation of the Forskolin-Induced Swelling Assay in Cystic Fibrosis Human Colon Organoids. STAR Protoc..

[143] Dekkers J.F., Gondra R.A.G., Kruisselbrink E., Vonk A.M., Janssens H.M., Groot K.M.D.W., Van Der Ent C.K., Beekman J.M. (2016). Optimal correction of distinct CFTR folding mutants in rectal cystic fibrosis organoids. Eur. Respir. J..

[144] Maule G., Casini A., Montagna C., Ramalho A.S., De Boeck K., Debyser Z., Carlon M.S., Petris G., Cereseto A. (2019). Allele specific repair of splicing mutations in cystic fibrosis through AsCas12a genome editing. Nat. Commun..

[145] Geurts M.H., de Poel E., Amatngalim G.D., Oka R., Meijers F.M., Kruisselbrink E., van Mourik P., Berkers G., de Winter-de Groot K.M., Michel S. (2020). CRISPRBased Adenine Editors Correct Nonsense Mutations in a Cystic Fibrosis Organoid Biobank. Cell Stem Cell.

[146] Silva I.A.L., Railean V., Duarte A., Amaral M.D. (2021). Personalized Medicine Based on Nasal Epithelial Cells: Comparative Studies with Rectal Biopsies and Intestinal Organoids. J. Pers. Med..

[147] Collaco J.M., Blackman S.M., Raraigh K.S., Corvol H., Rommens J.M., Pace R.G., Boelle P.Y., McGready J., Sosnay P.R., Strug L.J. (2016). Sources of Variation in Sweat Chloride Measurements in Cystic Fibrosis. Am. J. Respir. Crit. Care Med..

[148] de Winter-de Groot K.M., Janssens H.M., Van Uum R.T., Dekkers J.F., Berkers G., Vonk A., Kruisselbrink E., Oppelaar H., Vries R., Clevers H. (2018). Stratifying infants with cystic fibrosis for disease severity using intestinal organoid swelling as a biomarker of CFTR function. Eur. Respir. J..

[149] Rowe S.M., Accurso F., Clancy J.P. (2007). Detection of Cystic Fibrosis Transmembrane Conductance Regulator Activity in Early-Phase Clinical Trials. Proc. Am. Thorac. Soc..

[150] Kyrilli S., Henry T., Wilschanski M., Fajac I., Davies J.C., Jais J.P., Sermet-Gaudelus I. (2020). Insights into the variability of nasal potential difference, a biomarker of CFTR activity. J. Cyst. Fibros..

[151] Muilwijk D., de Poel E., van Mourik P., Suen S.W.F., Vonk A.M., Brunsveld J.E., Kruisselbrink E., Oppelaar H., Hagemeijer M.C., Berkers G. (2022). Forskolin-induced Organoid Swelling is Associated with Long-term CF Disease Progression. Eur. Respir. J..

[152] de Poel E., Spelier S., Suen S.W.F., Kruisselbrink E., Graeber S.Y., Mall M.A., Weersink E.J.M., van der Eerden M.M., Koppelman G.H., van der Ent C.K. (2022). Functional Restoration of CFTR Nonsense Mutations in Intestinal Organoids. J. Cyst. Fibros..

[153] de Winter–de Groot K.M., Berkers G., Marck–van der Wilt R.E.P., van der Meer R., Vonk A., Dekkers J.F., Geerdink M., Michel S., Kruisselbrink E., Vries R. (2020). Forskolin-induced swelling of intestinal organoids correlates with disease severity in adults with cystic fibrosis and homozygous F508del mutations. J. Cyst. Fibros..

[154] Ramalho A.S., Furstova E., Vonk A.M., Ferrante M., Verfailli C., Dupont L., Boon M., Proesmans M., Beekma J.M., Sarouk I. (2021). Correction of CFTR function in intestinal organoids to guide treatment of cystic fibrosis. Eur. Respir. J..

[155] Aalbers B.L., Brunsveld J.E., van der Ent C.K., van den Eijnden J.C., Beekman J.M., Heijerman H.G.M. (2022). Forskolin induced swelling (FIS) assay in intestinal organoids to guide eligibility for compassionate use treatment in a CF patient with a rare genotype. J. Cyst. Fibros..

[156] Graeber S.Y., van Mourik P., Vonk A.M., Kruisselbrink E., Hirtz S., van der Ent C.K., Mall M.A., Beekman J.M. (2020). Comparison of Organoid Swelling and In Vivo Biomarkers of CFTR Function to Determine Effects of Lumacaftor–Ivacaftor in Patients with Cystic Fibrosis Homozygous for the F508del Mutation. Am. J. Respir. Crit. Care Med..

[157] Zomer-van Ommen D.D., de Poel E., Kruisselbrink E., Oppelaar H., Vonk A.M., Janssens H.M., van der Ent C.K., Hagemeijer M.C., Beekman J.M. (2018). Comparison of ex vivo and in vitro intestinal cystic fibrosis models to measure CFTR-dependent ion channel activity. J. Cyst. Fibros..

[158] Ciciriello F., Bijvelds M.J.C., Alghisi F., Meijsen K.F., Cristiani L., Sorio C., Melotti P., Fiocchi A.G., Lucidi V., De Jonge H.R. (2022). Theratyping of the Rare CFTR Variants E193K and R334W in Rectal Organoid-Derived Epithelial Monolayers. J. Pers. Med..

[159] Ferrera L., Baroni D., Moran O. (2019). Lumacaftor-rescued F508del-CFTR has a modified bicarbonate permeability. J. Cyst. Fibros..

[160] Fiore M., Picco C., Moran O. (2020). Correctors modify the bicarbonate permeability of F508del-CFTR. Sci. Rep..

[161] Foulke-Abel J., In J., Yin J., Zachos N.C., Kovbasnjuk O., Estes M.K., de Jonge H., Donowitz M. (2016). Human Enteroids as a Model of Upper Small Intestinal Ion Transport Physiology and Pathophysiology. Gastroenterology.

[162] Al Abbar A., Ngai S.C., Nograles N., Alhaji S.Y., Abdullah S. (2020). Induced Pluripotent Stem Cells: Reprogramming Platforms and Applications in Cell Replacement Therapy. BioResearch Open Access.

[163] Ensinck M., Mottais A., Detry C., Leal T., Carlon M.S. (2021). On the Corner of Models and Cure: Gene Editing in Cystic Fibrosis. Front. Pharmacol..

[164] Wong A.P., Bear C.E., Chin S., Pasceri P., Thompson T.O., Huan L.J., Ratjen F., Ellis J., Rossant J. (2012). Directed differentiation of human pluripotent stem cells into mature airway epithelia expressing functional CFTR protein. Nat. Biotechnol..

[165] McCauley K.B., Hawkins F., Serra M., Thomas D.C., Jacob A., Kotton D.N. (2017). Efficient Derivation of Functional Human Airway Epithelium from Pluripotent Stem Cells via Temporal Regulation of Wnt Signaling. Cell Stem Cell.

[166] Huang S.X.L., Islam M.N., O’Neill J., Hu Z., Yang Y.G., Chen Y.W., Mumau M., Green M.D., Vunjak-Novakovic G., Bhattacharya J. (2014). Efficient generation of lung and airway epithelial cells from human pluripotent stem cells. Nat. Biotechnol..

[167] Firth A.L., Dargitz C.T., Qualls S.J., Menon T., Wright R., Singer O., Gage F.H., Khanna A., Verma I.M. (2014). Generation of multiciliated cells in functional airway epithelia from human induced pluripotent stem cells. Proc. Natl. Acad. Sci. USA..

[168] Hawkins F., Kramer P., Jacob A., Driver I., Thomas D.C., McCauley K.B., Skvir N., Crane A.M., Kurmann A.A., Hollenberg A.N. (2017). Prospective isolation of NKX2-1–expressing human lung progenitors derived from pluripotent stem cells. J. Clin. Investig..

[169] Merkert S., Schubert M., Olmer R., Engels L., Radetzki S., Veltman M., Scholte B.J., Zöllner J., Pedemonte N., Galietta L.J.V. (2019). High-Throughput Screening for Modulators of CFTR Activity Based on Genetically Engineered Cystic Fibrosis Disease-Specific iPSCs. Stem Cell Rep..

[170] Ahmadi S., Bozoky Z., Di Paola M., Xia S., Li C., Wong A.P., Wellhauser L., Molinski S.V., Ip W., Ouyang H. (2017). Phenotypic profiling of CFTR modulators in patient-derived respiratory epithelia. NPJ Genom. Med..

[171] Crane A.M., Kramer P., Bui J.H., Chung W.J., Li X.S., Gonzalez-Garay M.L., Hawkins F., Liao W., Mora D., Choi S. (2015). Targeted Correction and Restored Function of the CFTR Gene in Cystic Fibrosis Induced Pluripotent Stem Cells. Stem Cell Rep..

[172] Jiang J.X., Wellhauser L., Laselva O., Utkina I., Bozoky Z., Gunawardena T., Ngan Z., Xia S., Di Paola M., Eckford P.D.W. (2021). A new platform for high-throughput therapy testing on iPSC-derived lung progenitor cells from cystic fibrosis patients. Stem Cell Rep..

[173] Berical A., Lee R.E., Lu J., Beermann M.L., Le Suer J.A., Mithal A., Thomas D., Ranallo N., Peasley M., Stuffer A. (2022). A multimodal iPSC platform for cystic fibrosis drug testing. Nat. Commun..

[174] Ngan S.Y., Quach H.T., Laselva O., Huang E.N., Mangos M., Xia S., Bear C.E., Wong A.P. (2022). Stage-Specific Generation of Human Pluripotent Stem Cell Derived Lung Models to Measure CFTR Function. Curr. Protoc..

[175] Erwood S., Laselva O., Bily T.M., Brewer R.A., Rutherford A.H., Bear C.E., Ivakine E.A. (2020). Allele-Specific Prevention of Nonsense-Mediated Decay in Cystic Fibrosis Using Homology-Independent Genome Editing. Mol. Ther. Methods Clin. Dev..

[176] Smirnikhina S.A., Kondrateva E.V., Adilgereeva E.P., Anuchina A.A., Zaynitdinova M.I., Slesarenko Y.S., Ershova A.S., Ustinov K.D., Yasinovsky M.I., Amelina E.L. (2020). P.F508del editing in cells from cystic fibrosis patients. PLoS ONE.

[177] Ruan J., Hirai H., Yang D., Ma L., Hou X., Jiang H., Wei H., Rajagopalan C., Mou H., Wang G. (2019). Efficient Gene Editing at Major CFTR Mutation Loci. Mol. Ther. Nucleic Acids.

[178] Firth A.L., Menon T., Parker G.S., Qualls S.J., Lewis B.M., Ke E., Dargitz C.T., Wright R., Khanna A., Gage F.H. (2015). Functional Gene Correction for Cystic Fibrosis in Lung Epithelial Cells Generated from Patient iPSCs. Cell Rep..

[179] Suzuki S., Sargent R.G., Illek B., Fischer H., Esmaeili-Shandiz A., Yezzi M.J., Lee A., Yang Y., Kim S., Renz P. (2016). TALENs Facilitate Single-step Seamless SDF Correction of F508del CFTR in Airway Epithelial Submucosal Gland Cell-derived CF-iPSCs. Mol. Ther. Nucleic Acids.

[180] Spence J.R., Mayhew C.N., Rankin S.A., Kuhar M.F., Vallance J.E., Tolle K., Hoskins E.E., Kalinichenko V.V., Wells S.I., Zorn A.M. (2011). Directed differentiation of human pluripotent stem cells into intestinal tissue in vitro. Nature.

[181] Merkert S., Bednarski C., Göhring G., Cathomen T., Martin U. (2017). Generation of a gene-corrected isogenic control iPSC line from cystic fibrosis patient-specific iPSCs homozygous for p.Phe508del mutation mediated by TALENs and ssODN. Stem Cell Res..

[182] Mithal A., Capilla A., Heinze D., Berical A., Villacorta-Martin C., Vedaie M., Jacob A., Abo K., Szymaniak A., Peasley M. (2020). Generation of mesenchyme free intestinal organoids from human induced pluripotent stem cells. Nat. Commun..

[183] Watson C.L., Mahe M.M., Múnera J., Howell J.C., Sundaram N., Poling H.M., Schweitzer J.I., Vallance J.E., Mayhew C.N., Sun Y. (2014). An in vivo model of human small intestine using pluripotent stem cells. Nat. Med..

[184] Xia S., Bozóky Z., Di Paola M., Laselva O., Ahmadi S., Jiang J.X., Pitstick A.L., Jiang C., Rotin D., Mayhew C.N. (2021). High-Throughput Functional Analysis of CFTR and Other Apically Localized Proteins in iPSCDerived Human Intestinal Organoids. Cells.

[185] Hohwieler M., Illing A., Hermann P.C., Mayer T., Stockmann M., Perkhofer L., Eiseler T., Antony J.S., Müller M., Renz S. (2017). Human pluripotent stem cell-derived acinar/ductal organoids generate human pancreas upon orthotopic transplantation and allow disease modelling. Gut.

[186] Ogawa M., Ogawa S., E Bear C., Ahmadi S., Chin S., Li B., Grompe M., Keller G., Kamath B.M., Ghanekar A. (2015). Directed differentiation of cholangiocytes from human pluripotent stem cells. Nat. Biotechnol..

[187] Dianat N., Dubois-Pot-Schneider H., Steichen C., Desterke C., Leclerc P., Raveux A., Combettes L., Weber A., Corlu A., Dubart-Kupperschmitt A. (2014). Generation of functional cholangiocyte-like cells from human pluripotent stem cells and HepaRG cells. Hepatology.

[188] Fiorotto R., Amenduni M., Mariotti V., Fabris L., Spirli C., Strazzabosco M. (2018). Src kinase inhibition reduces inflammatory and cytoskeletal changes in ΔF508 human cholangiocytes and improves cystic fibrosis transmembrane conductance regulator correctors efficacy. Hepatology.

[189] Ogawa M., Jiang J.X., Xia S., Yang D., Ding A., Laselva O., Hernandez M., Cui C., Higuchi Y., Suemizu H. (2021). Generation of functional ciliated cholangiocytes from human pluripotent stem cells. Nat. Commun..

[190] Huh D., Matthews B.D., Mammoto A., Montoya-Zavala M., Yuan Hsin H., Ingber D.E. (2010). Reconstituting Organ-Level Lung Functions on a Chip. Science.

[191] Wu Q., Liu J., Wang X., Feng L., Wu J., Zhu X., Wen W., Gong X. (2020). Organ-on-a-chip: Recent breakthroughs and future prospects. Biomed. Eng. Online.

[192] Konar D., Devarasetty M., Yildiz D.V., Atala A., Murphy S.V. (2016). Lung-On-AChip Technologies for Disease Modeling and Drug Development. Biomed. Eng. Comput. Biol..

[193] Mejías J.C., Nelson M.R., Liseth O., Roy K. (2020). A 96-well format microvascularized human lung-on-a-chip platform for microphysiological modeling of fibrotic diseases. Lab Chip.

[194] Plebani R., Potla R., Soong M., Bai H., Izadifar Z., Jiang A., Travis R.N., Belgur C., Dinis A., Cartwright M.J. (2021). Modeling pulmonary cystic fibrosis in a human lung airway-on-a-chip: Cystic fibrosis airway chip. J. Cyst. Fibros..

[195] Benam K.H., Villenave R., Lucchesi C., Varone A., Hubeau C., Lee H.H., E Alves S., Salmon M., Ferrante T.C., Weaver J.C. (2015). Small airway-on-a-chip enables analysis of human lung inflammation and drug responses in vitro. Nat. Methods.

[196] Nawroth J.C., Lucchesi C., Cheng D., Shukla A., Ngyuen J., Shroff T., Varone A., Karalis K., Lee H.H., Alves S. (2020). A Microengineered Airway Lung Chip Models Key Features of Viral-induced Exacerbation of Asthma. Am. J. Respir. Cell Mol. Biol..

[197] Lavelle G.M., White M.M., Browne N., McElvaney N.G., Reeves E.P. (2016). Animal Models of Cystic Fibrosis Pathology: Phenotypic Parallels and Divergences. BioMed Res. Int..

[198] Semaniakou A., Croll R.P., Chappe V. (2019). Animal Models in the Pathophysiology of Cystic Fibrosis. Front. Pharmacol..

[199] Shik Mun K., Arora K., Huang Y., Yang F., Yarlagadda S., Ramananda Y., Abu-El-Haija M., Palermo J.J., Appakalai B.N., Nathan J.D. (2019). Patient-derived pancreas-on-a-chip to model cystic fibrosis-related disorders. Nat. Commun..

[200] Fawcett L.K., Wakefield C.E., Sivam S., Middleton P.G., Wark P., Widger J., Jaffe A., Waters S.A. (2021). Avatar acceptability: Views from the Australian Cystic Fibrosis community on the use of personalised organoid technology to guide treatment decisions. ERJ Open Res..

